# PcNRAMP1 Enhances Cadmium Uptake and Accumulation in *Populus* × *canescens*

**DOI:** 10.3390/ijms23147593

**Published:** 2022-07-08

**Authors:** Wenjian Yu, Shurong Deng, Xin Chen, Yao Cheng, Zhuorong Li, Jiangting Wu, Dongyue Zhu, Jing Zhou, Yuan Cao, Payam Fayyaz, Wenguang Shi, Zhibin Luo

**Affiliations:** 1 State Key Laboratory of Tree Genetics and Breeding, Key Laboratory of Silviculture of the National Forestry and Grassland Administration, Research Institute of Forestry, Chinese Academy of Forestry, Beijing 100091, China; yuwj@caf.ac.cn (W.Y.); dengsr@caf.ac.cn (S.D.); chenxincaf@caf.ac.cn (X.C.); chengy@caf.ac.cn (Y.C.); lizhuorong119lky@caf.ac.cn (Z.L.); wujt@caf.ac.cn (J.W.); zhudy@caf.ac.cn (D.Z.); gaha2008@caf.ac.cn (J.Z.); ycao2012@caf.ac.cn (Y.C.); 2 Forest, Range and Watershed Management Department, Agriculture and Natural Resources Faculty, Yasouj University, Yasuj 75919-63179, Iran; pfayyaz@yu.ac.ir

**Keywords:** poplar, cadmium, NRAMP, Cd^2+^ flux, conserved amino acid residues

## Abstract

Poplars are proposed for the phytoremediation of heavy metal (HM) polluted soil. Characterization of genes involved in HM uptake and accumulation in poplars is crucial for improving the phytoremediation efficiency. Here, *Natural Resistance-Associated Macrophage Protein* 1 (*NRAMP1*) encoding a transporter involved in cadmium (Cd) uptake and transport was functionally characterized in *Populus* × *canescens*. Eight putative *PcNRAMPs* were identified in the poplar genome and most of them were primarily expressed in the roots. The expression of *PcNRAMP1* was induced in Cd-exposed roots and it encoded a plasma membrane-localized protein. PcNRAMP1 showed transport activity for Cd^2+^ when expressed in yeast. The *PcNRAMP1*-overexpressed poplars enhanced net Cd^2+^ influxes by 39–52% in the roots and Cd accumulation by 25–29% in aerial parts compared to the wildtype (WT). However, Cd-induced biomass decreases were similar between the transgenics and WT. Further analysis displayed that the two amino acid residues of PcNRAMP1, i.e., M236 and P405, play pivotal roles in regulating its transport activity for Cd^2+^. These results suggest that PcNRAMP1 is a plasma membrane-localized transporter involved in Cd uptake and transporting Cd from the roots to aerial tissues, and that the conserved residues in PcNRAMP1 are essential for its Cd transport activity in poplars.

## 1. Introduction

Cadmium (Cd, hereafter it can also be considered as Cd^2+^ in the relevant context) is a highly toxic heavy metal (HM) for most organisms. Due to anthropogenic activities, Cd pollution has become a serious problem worldwide. Cd in soil can eventually enter into the human body through the food chain, leading to serious health problems [1]. Phytoremediation has been proposed as an environment-friendly and cost-effective approach to remediate Cd-contaminated soil [2,3]. The key to success in phytoremediation is to find plants with great capacities to take up Cd from the soil and transport it to aerial tissues which can be easily harvested [4]. Several Cd hyperaccumulating herbaceous plants, such as *Noccaea* (*Thlaspi*) *caerulescens*, *Sedum alfredii* and *Solanum nigrum*, have been identified [5,6,7]. However, Cd accumulation is limited in these herbaceous plants, due to their low biomass production. Alternatively, fast growing woody plants, including *Populus* species, have been proposed for the phytoremediation [8]. *Populus* species can produce a large amount of biomass and possess a deep root system. Previous studies have demonstrated that some *Populus* species can accumulate relatively high amounts of Cd in aerial tissues [9,10]. Notably, the capacities for Cd uptake and accumulation can be further improved in poplars through genetic engineering [11].

Cd is often taken up by transporters involved in transporting of divalent cations of essential elements in the roots of plants. Several transporters, including the Natural Resistance-Associated Macrophage Proteins (NRAMPs), the Zinc-Iron regulated Proteins (ZIPs), the Heavy Metal ATPases (HMAs) and the ATP-Binding Cassette family proteins (ABCs), have been identified for Cd transport activities [12,13]. Among these transporters, NRAMPs play a pivotal role in Cd uptake and accumulation in plants. The molecular functions of several NRAMPs have been extensively investigated in some herbaceous plants [14]. In the genome of *Arabidopsis thaliana*, six *NRAMP* members have been identified [15]. Particularly, AtNRAMP1 is localized in the plasma membrane and highly expressed in the roots [16]. AtNRAMP1 acts as a transporter by taking up external Cd into the cytosol of cells. Additionally, AtNRAMP3, AtNRAMP4 and AtNRAMP6 are involved in Cd transport and sequestration in different organelles of plant cells [17,18,19]. In rice (*Oryza sative*), there are seven members of NRAMPs [20]. OsNRAMP1 is localized in the plasma membrane and overexpressing *OsNRAMP1* in rice results in more Cd uptake by the roots, which is then translocated to aerial tissues [20,21,22]. *OsNRAMP1* is mainly expressed in the roots and leaves and its expression is induced by Cd exposure [21]. In addition, OsNRAMP5 is also involved in Cd uptake and translocation [23,24]. Although great progress has been made in characterizing molecular functions of NRAMPs in herbaceous plants, little information is available about the functions of NRAMPs in woody plants.

There are ten to twelve transmembrane segments (TMSs) in NRAMPs [25]. These TMSs play pivotal roles in the maintenance of the three-dimensional conformation of NRAMPs [25]. It has been demonstrated that alternating access of the co-substrate binding sites toward either side of the membrane is mediated by the symmetric interface that acts as a switch for conformational exchange between two structural blocks, a helical bundle (TMS1, 2, 6, 7) and a hash motif (TMS3, 4, 8, 9) of NRAMPs [25]. Specifically, TMS5 and TMS10 are involved in embracing the bundle and gating the transport mechanism [25]. TMS1, TMS6, TMS3 and TMS8, at the central positions, contribute forming the co-substrate-binding sites for metal translocation [25]. Moreover, the conserved amino acid residues in the TMS are critical for the functions of NRAMPs [26]. Mutations G119A and Q126D in the mammalian transporter DCT1 (a NRAMP) lead to almost complete inhibition of the activity of metal ion uptake [26]. In *A. thaliana*, mutations at the sites of L67, E401 and F413 in AtNRAMP4 result in changes in metal selectivity of the transporter [27]. Recently, it has been found that the conserved residues in the motif B of OsNRAT1 and FeNRAMP5 are essential for the selectivity of Al and Mn, respectively [28,29]. Currently, however, no information is available on which amino acid residues are essential for the metal uptake activity of NRAMPs in woody plants.

*Populus* species are ideal woody plants for the phytoremediation of Cd-contaminated soil. Genes encoding several transporters, such as NRAMPs, ZIPs and HMAs, have been identified in the genome of *P. trichocarpa* [30]. A few studies have attempted to characterize the molecular functions of several transporters involved in the transport of Cd and other metals in poplars [31,32,33]. However, the molecular functions of NRAMPs in *Populus* species remain elusive. Previously, we found that *Populus* × *canescens* can accumulate relatively higher levels of Cd in the root, wood, bark and leaf tissues in comparison with other poplar species [9]. Moreover, the expression levels of several *NRAMPs* have been induced in *P*. × *canescens* due to the presence of Cd [34]. It is likely that NRAMPs play pivotal roles in Cd uptake and accumulation in *P*. × *canescens*. To investigate the molecular functions of *NRAMPs* involved in Cd uptake and accumulation in poplars, we first systematically identified *NRAMP* members in the genome of *P*. × *canescens*. Then, based on the expression pattern of the identified *PcNRAMPs*, *PcNRAMP1* was selected to be functionally characterized for its involvement in Cd uptake and transport. Particularly relevant is the fact that the amino acid residues in TMS, which are essential for Cd transport activity, were identified in *PcNRAMP1*. The results of this study will provide important insights into the phytoremediation of HM-polluted soil by using genetically modified woody plants.

## 2. Results

### 2.1. Identification and Phylogenetic Tree of NRAMP Genes

**Figure 1:**
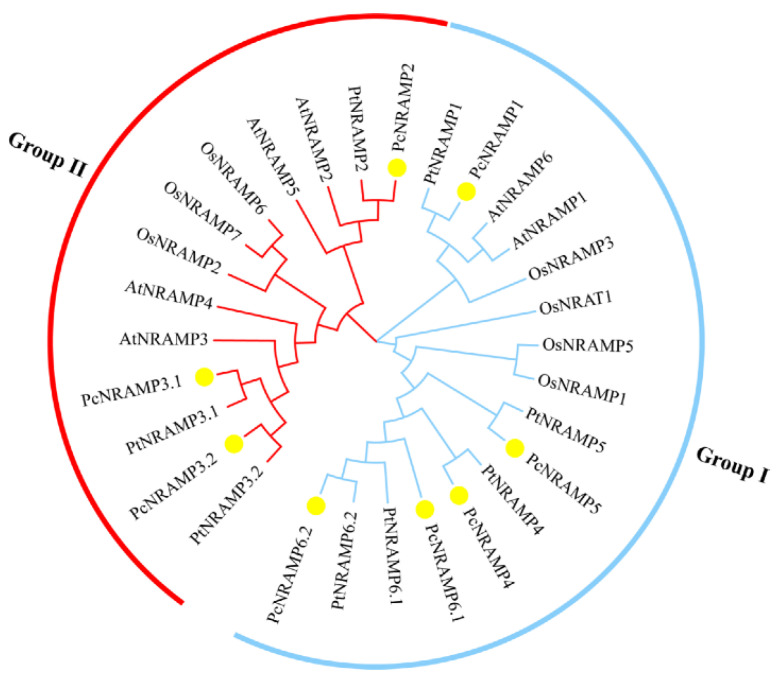
Phylogenetic analysis of NRAMP proteins in *P*. × *canescens* (Pc), *P. trichocarpa* (Pt), *A. thaliana* (At) and *O. sativa* (Os). Blue and red branches represent group I and II, respectively. PcNRAMP proteins are marked with yellow filled circles. Accession numbers of NRAMP proteins are listed in Table S2.

Eight putative *NRAMP* genes encoding NRAMP1, NRAMP2, NRAMP3.1/3.2, NRAMP4, NRAMP5 and NRAMP6.1/6.2 with NRAMP domain (PF01566) were identified in the genome of *P*. × *canescens* (Figure 1, Table S2). All identified PcNRAMPs were named according to their homologues in *A. thaliana* and *O. sativa* (Figure 1, Table S2). The phylogenetic analysis was conducted using NRAMPs from *P*. × *canescens*, *P*. *trichocarpa*, *A. thaliana* and *O. sativa*. The tested NRAMPs from these four species were divided into two groups, i.e., group I and II (Figure 1, Table S2). Five PcNRAMP members, i.e., PcNRAMP1, PcNRAMP4, PcNRAMP5, PcNRAMP6.1 and PcNRAMP6.2, were included in group I, and PcNRAMP2, PcNRAMP3.1 and PcNRAMP3.2 belonged to group II (Figure 1, Table S2). The PcNRAMP proteins had 500 to 585 amino acid residues in length, 54.64 to 63.95 kDa in molecular weight, and 4.93 to 8.42 in theoretical isoelectric point (Table S3). The PcNRAMP proteins contained 10 to 12 transmembrane helices (Table S3).

### 2.2. Expression Patterns of the PcNRAMP Genes

**Figure 2:**
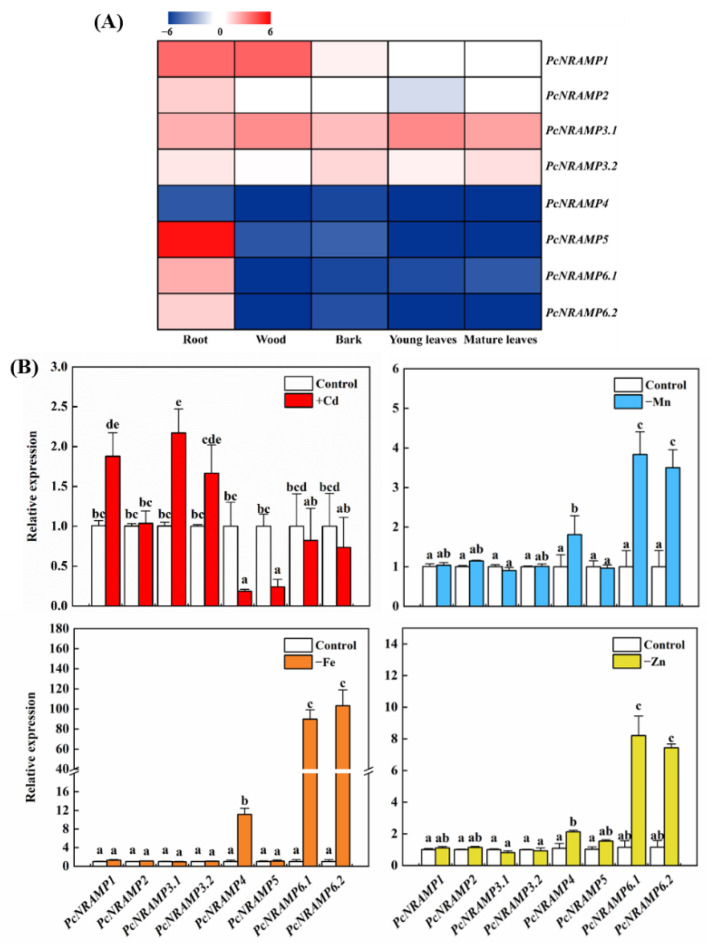
The expression profiles of *PcNRAMP* genes. The expression levels of *PcNRAMP* genes in different tissues without Cd exposure (**A**) and in the roots (**B**) of *P*. × *canescens* supplied with one-fourth Hoagland solution in combination with either 0 (−Cd) or 100 (+Cd) μM CdCl_2_, and the deficiency in one of Mn (−Mn), Fe (−Fe) and Zn (−Zn) for 7 days. Bars indicate means ± SE (n = 4). Different letters on the bars indicate significant differences in the relative expression levels among different *PcNRAMP* genes. In panel (**A**), the expression level of each *PcNRAMP* in different tissues was compared with the median of all genes, and the fold change in the expression level of each *PcNRAMP* was calculated based on the log2 value of its expression level. In panel (**B**), for each gene, the expression level was set to 1 in the roots of control plants, and the corresponding fold changes were calculated under other treatments.

The expression patterns of *PcNRAMP* genes were analyzed in different tissues without Cd exposure and in the roots of *P*. × *canescens* in response to metal treatments (Figure 2). *PcNRAMP1* was highly expressed in the root and wood tissues of *P*. × *canescens*, and its expression levels were very low in the other tissues (Figure 2A). The transcript levels of *PcNRAMP2* and *PcNRAMP3.2* were low in the examined tissues of *P*. × *canescens*, the mRNA levels of *PcNRAMP3.1* were relatively high in all examined tissues, and the expression levels of *PcNRAMP4*, *PcNRAMP6.1* and *PcNRAMP6.2* were extremely low in all tested tissues (Figure 2A). The mRNA level of *PcNRAMP5* was very high in the roots, but its transcript levels were extremely low in other examined tissues (Figure 2A). The mRNA levels of *PcNRAMP1*, *PcNRAMP3.1* and *PcNRAMP3.2* were upregulated, and the transcript levels of *PcNRAMP4* and *PcNRAMP5* were decreased in the roots of Cd-treated *P*. × *canescens* (Figure 2B). The expression levels of *PcNRAMP4*, *PcNRAMP6.1* and *PcNRAMP6.2* were markedly upregulated, and the transcript abundance of other *PcNRAMP* genes remained unaltered in the roots of *P*. × *canescens* in response to deficiency in one of the following: Mn, Fe and Zn (Figure 2B). These results have demonstrated that *PcNRAMP1* is mainly expressed in the roots and induced upon Cd exposure, suggesting that *PcNRAMP1* can play a critical role in Cd uptake and accumulation of *P*. × *canescens*. Thus, we investigated the spatial and temporal expression patterns of *PcNRAMP1* and its molecular functions in *P*. × *canescens* in response to Cd exposure.

The transcript levels of *PcNRAMP1* were upregulated in the roots after Cd exposure for 12 h and one week, respectively (Figure S2). In the bark and young leaves, the expression levels of *PcNRAMP1* were induced by Cd treatment in 12 h (Figure S2). The mRNA levels of *PcNRAMP1* were decreased in the wood exposed to Cd for 12 h and one week (Figure S2). The expression level of *PcNRAMP1* was decreased in the mature leaves treated with Cd for 12 h, but it was increased after Cd treatment for 72 h and one week (Figure S2). These results indicate that the expression of *PcNRAMP1* in the roots and mature leaves is more responsive to Cd exposure across all the time points examined in this study and the molecular regulation mechanisms underlying the temporal and spatial changes in the expression levels of *PcNRAMP1* deserve to be further exploited.

### 2.3. Subcellular Localization of PcNRAMP1

**Figure 3:**
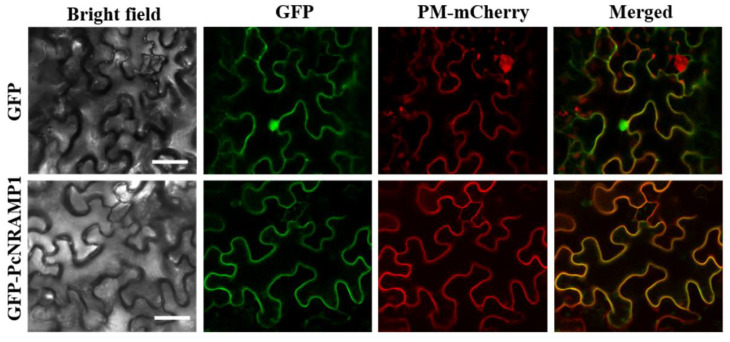
Subcellular localization of PcNRAMP1 in leaf epidermal cells of tobacco. The bright field, the green fluorescent protein (GFP) fluorescence, PM-mCherry and the merged images are shown. Scale bar = 20 μm.

To explore the subcellular localization of PcNRAMP1 proteins, we transiently co-expressed GFP-PcNRAMP1 fusion protein with a plasma membrane marker pMDC32-1A CAN2b-mCherry in epidermal cells of *N*. *benthamiana* leaves. The green fluorescence signal was co-localized with the signal of the plasma membrane marker (Figure 3), indicating that PcNRAMP1 is localized at the plasma membrane.

### 2.4. PcNRAMP1 Showed Transport Activities for Cd, Mn and Fe in Yeast

**Figure 4:**
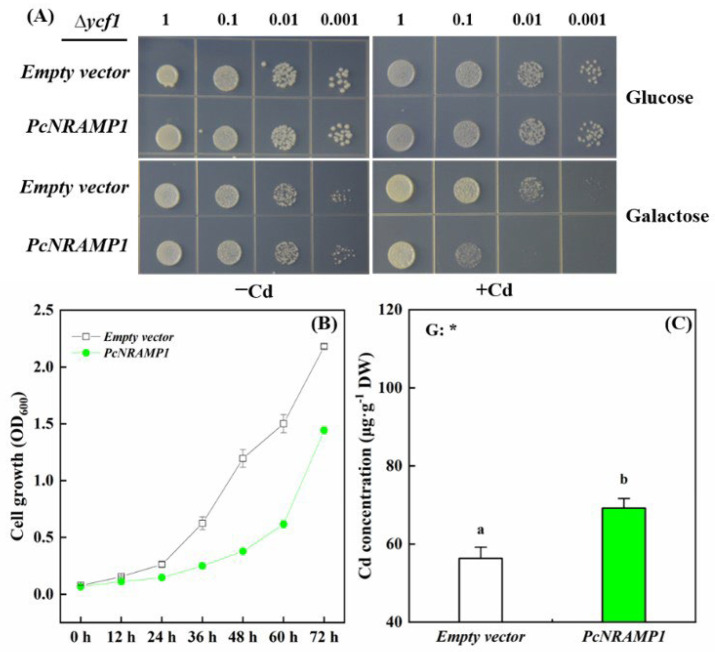
Cd transport activity assay of PcNRAMP1 in yeast. (**A**) The growth status of yeast mutant strain ∆*ycf1* defective in Cd uptake cells in which either empty vector or *PcNRAMP1* was transformed. The yeast cells diluted with OD_600_ nm of 1−0.001 were cultured in the plate with either glucose or galactose under either 0 (−Cd) or 30 (+Cd) μM CdCl_2_ for 4 days. (**B**) The cell growth dynamics under Cd exposure in 72 h. (**C**) The concentration of Cd in yeast cells. Bars indicate means ± SE (n = 4). Different letters on the bars indicate significant differences. *p*-value of the one-way ANOVA of genotypes (G) is also indicated. *: *p* < 0.05.

To investigate Cd transport activity of PcNRAMP1 in yeast, the full-length coding region of *PcNRAMP1* was cloned into the pYES2 vector, where the expression of *PcNRAMP1* was under the control of a glucose repressive and galactose inducible GAL1 promoter. The pYES2-*PcNRAMP1* and the empty vector (pYES2) were transformed to Cd-sensitive mutant yeast strain ∆*ycf1*, respectively. In the presence of glucose, the growth of yeast expressing either *PcNRAMP1* or the empty vector displayed no difference under the conditions of 0 and 30 μM Cd exposure (Figure 4A). In the presence of galactose, the growth of yeast expressing *PcNRAMP1* was similar to that expressing the empty vector under 0 μM Cd condition, but it was inhibited in comparison with that expressing the empty vector under 30 μM Cd condition (Figure 4A). This finding indicates that the sensitivity to Cd of ∆*ycf1* is enhanced by *PcNRAMP1* expression compared to the empty vector. The increased Cd sensitivity was further confirmed by the growth dynamics of ∆*ycf1* expressing either *PcNRAMP1* or the empty vector in 72 h under 30 μM Cd condition (Figure 4B). Moreover, the yeast expressing *PcNRAMP1* accumulated greater Cd in comparison with the one expressing the empty vector (Figure 4C). These results indicate that PcNRAMP1 is able to take up Cd from the medium into the yeast cells.

Similarly, to examine Mn and Fe transport activities of PcNRAMP1 in yeast, the full-length coding region of *PcNRAMP1* was cloned into the pYES2 vector. Both pYES2-*PcNRAMP1* and the empty vector (pYES2) were transformed to mutant yeast strains ∆*pmr1* and ∆*ccc1*, respectively. In the presence of galactose, the growth of yeast expressing *PcNRAMP1* was similar to that expressing the empty vector under the control conditions of Mn or Fe treatments, but it was markedly repressed in comparison with that expressing the empty vector under either 1 mM Mn or 8 mM Fe conditions (Figure S3). These results suggest that the sensitivity to Mn of ∆*pmr1* and to Fe of ∆*ccc1* is enhanced by *PcNRAMP1* expression, compared to the empty vector.

### 2.5. Functional Analysis of PcNRAMP1 in P. × canescens

**Figure 5:**
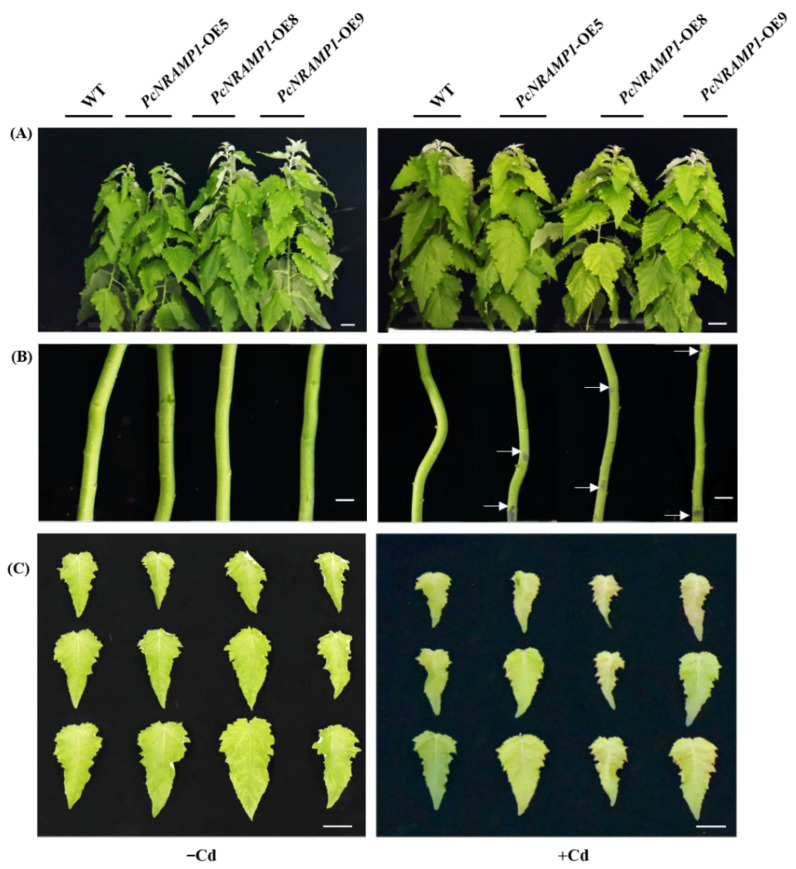
Growth performance of WT and transgenic lines in response to Cd stress. (**A**) *PcNRAMP1*-OE5, *PcNRAMP1*-OE8, *PcNRAMP1*-OE9 of *P*. × *canescens* treated with either 0 (−Cd) or 100 (+Cd) μM CdCl_2_ for 2 weeks. (**B**,**C**) The phenotypes of the stems and the leaves (LPI = 7–9) of WT and transgenic lines demonstrating Cd toxicity. The arrows in panel B point to black spots. The bars in panels (**A**–**C**) indicate 5, 1 and 4 cm, respectively.

To confirm Cd transport activity of PcNRAMP1 in *P*. × *canescens*, *35S*::*PcNRAMP1* was transformed to *P*. × *canescens*. Ten transgenic lines were obtained and confirmed by PCR (Figure S4A). The three transgenic lines with the highest expression levels of *PcNRAMP1* (*PcNRAMP1*-OE5, *PcNRAMP1*-OE8 and *PcNRAMP1*-OE9) were selected and cultured in hydroponics (Figure S4B). The growth rate of the transgenic poplars was similar to that of WT (Figure S4B). However, interveinal chlorosis in mature leaves was observed in transgenic plants and no such phenotype was found in WT plants (Figure S4C). The growth performance of WT and transgenic poplars treated with 100 μM Cd was also observed (Figure 5A). Notably, black spots on the stems and red speckles on the leaves were observed in *PcNRAMP1*-overexpressed *P*. × *canescens* exposed to Cd, but no such symptoms were found in Cd-treated WT plants (Figure 5B,C). The photosynthetic characteristics were similar in WT and transgenic *P*. × *canescens* without Cd exposure (Figure S5A). The photosynthetic rates (*A*) were significantly decreased in WT and transgenic poplars exposed to Cd in comparison with those of poplars treated with 0 μM Cd, and more reductions in *A* were observed in transgenics than in WT plants (Figure S5A). In line with interveinal chlorosis in mature leaves of transgenic poplars, the concentrations of chlorophyll a and b tended to decrease in *PcNRAMP1*-overexpressed plants without Cd treatment (Figure S5B), which is probably associated with the decreases in the concentrations of magnesium (Mg) in the leaves of transgenic poplars without Cd exposure (See below). The concentrations of chlorophylls were reduced in the mature leaves of WT and transgenic plants exposed to Cd in comparison with those without Cd treatment, and more decreases in concentrations of chlorophyll a and b were detected in transgenics than in WT poplars with Cd exposure (Figure S5B). No difference was observed in the biomass of WT and transgenic poplars treated with 0 μM Cd (Table S4). The biomass was markedly reduced in WT and transgenic poplars exposed to Cd in comparison with those of the poplars without Cd treatment, and Cd-induced reductions in biomass were similar between WT and transgenic poplars (Table S4).

**Figure 6:**
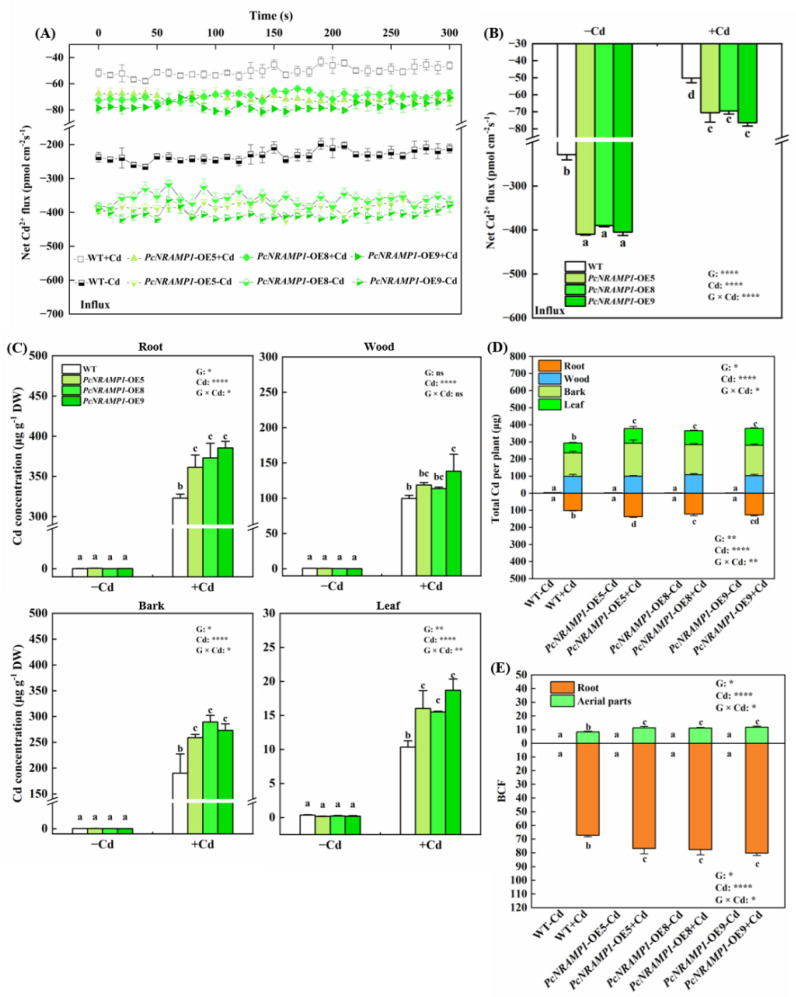
The net Cd^2+^ fluxes, metal concentration, Cd amounts, Cd bio-concentration factor (BCF) of WT and transgenic lines. (**A**,**B**) Net Cd^2+^ fluxes and the mean fluxes of Cd^2+^ in the roots, (**C**–**E**) Cd concentrations and amounts in different tissues and bio-concentration factor of WT and transgenic lines (*PcNRAMP1*-OE5, *PcNRAMP1*-OE8, *PcNRAMP1*-OE9) of *P*. × *canescens* treated with either 0 (−Cd) or 100 (+Cd) μM CdCl_2_ for 2 weeks. Data indicate means ± SE (n = 4). Different letters on the bars indicate significant differences between the treatments. *p*-values of the two-way ANOVAs of genotype (G), Cd and their interactions (G × Cd) are also indicated. *: *p* < 0.05; **: *p* < 0.01; ***: *p* < 0.001; ****: *p* < 0.0001; ns: not significant.

To find out the position along the root apex region where the maximal net Cd^2+^ influx occurs, we measured the net Cd^2+^ flux in various root zones from the root apex of WT and transgenic lines (Figure S1B). It turned out that the position with the maximal net Cd^2+^ influx was ca. at 600 μm from the tips of the first order roots of WT and transgenic poplars (Figure S1B). To further investigate the role of PcNRAMP1 in Cd^2+^ transport, we monitored the net Cd^2+^ fluxes at 600 μm from the root tips of fine roots of WT and transgenic poplars (Figure 6A,B). The net Cd^2+^ influxes of WT and transgenic poplars without Cd treatment ranged from −409.4 to −228.6 pmol cm^−2^ s^−1^, and the net Cd^2+^ influxes in the fine roots of three transgenic lines were 70.5 to 79.1% more rapid, compared to that in WT (Figure 6A,B). After 100 μM Cd exposure for two weeks, the net Cd^2+^ influxes of WT and three transgenic lines varied from −76.4 to −50.2 pmol cm^−2^ s^−1^, and the net Cd^2+^ influxes in the roots of the transgenic lines were 38.5 to 52.3% faster in comparison with that in WT plants (Figure 6A,B).

To examine Cd accumulation in poplars, Cd concentrations were determined in the root, wood, bark and leaf tissues of WT and transgenic lines (Figure 6C). No Cd was detected in WT and transgenics without Cd exposure (Figure 6C). Cd concentrations in the root, wood, bark and leaf tissues of transgenics were higher by 15.6, 24.1, 43.9 and 61.8%, respectively, than in the corresponding parts of WT plants under 100 μM Cd condition (Figure 6C). Cd concentrations were significantly increased in WT and transgenic plants exposed to Cd in comparison with those without Cd treatment (Figure 6C). Total Cd amounts in the roots and aerial parts of transgenics were greater by 27.0 to 27.6% than those of WT plants (Figure 6D). No Cd accumulation was detected in the roots and aerial parts of WT and transgenics without Cd exposure (Figure 6D). The BCFs were significantly higher in the roots and aerial parts of transgenic plants than in those of WT plants (Figure 6E).

Concentrations of some metal nutrients, including Mn, Fe, Zn, Ca and Mg, were also determined in the root, wood, bark and leaf tissues of WT and transgenic poplars (Table S5). Mn concentrations in different tissues of transgenic lines were higher than those of WT plants without Cd exposure, but no such differences were found between WT and transgenics after Cd exposure (Table S5). Mn concentrations of WT and transgenic lines with Cd exposure were significantly decreased in comparison with those without Cd treatment (Table S5). Consistently, Fe concentrations in different tissues of transgenic lines were greater than those of WT plants without Cd treatment (Table S5). Under Cd exposure condition, Fe levels in the roots of transgenics were higher in comparison with those of WT, and Fe concentrations were similar in the wood and bark tissues between WT and transgenic poplars, but Fe levels in the leaves of transgenic lines were lower than those of WT plants (Table S5). Fe concentrations were significantly greater in the Cd-exposed roots of WT and transgenic lines than those without Cd treatment, and Cd exposure resulted in unchanged Fe levels in the wood, bark and leaf tissues of WT plants, but Cd treatment led to decreased Fe concentrations in those tissues of transgenic lines (Table S5). Similar to Mn, Zn concentrations in the analyzed tissues of transgenic poplars were significantly higher than those of WT plants under 0 µM Cd condition (Table S5). In most cases, Zn levels were lower in the analyzed tissues of WT and transgenic poplars with Cd exposure than those without Cd treatment (Table S5). Ca concentrations were lower in the roots of transgenic plants than those of WT poplars, irrespective of Cd treatments, but the opposite was true in the wood (Table S5). Ca levels were higher in the roots of WT and transgenic plants treated with Cd in comparison with those without Cd exposure, but the opposite was true in the aerial tissues (Table S5). In most cases, Mg concentrations were lower in the root, bark and leaf tissues of transgenic poplars than those of WT plants, and Mg levels were lower in the root and leaf tissues of WT and transgenic poplars exposed to Cd in comparison with those without Cd treatment (Table S5).

### 2.6. Transport Activities of Metal Ions of Mutated PcNRAMP1 in Yeast

**Figure 7:**
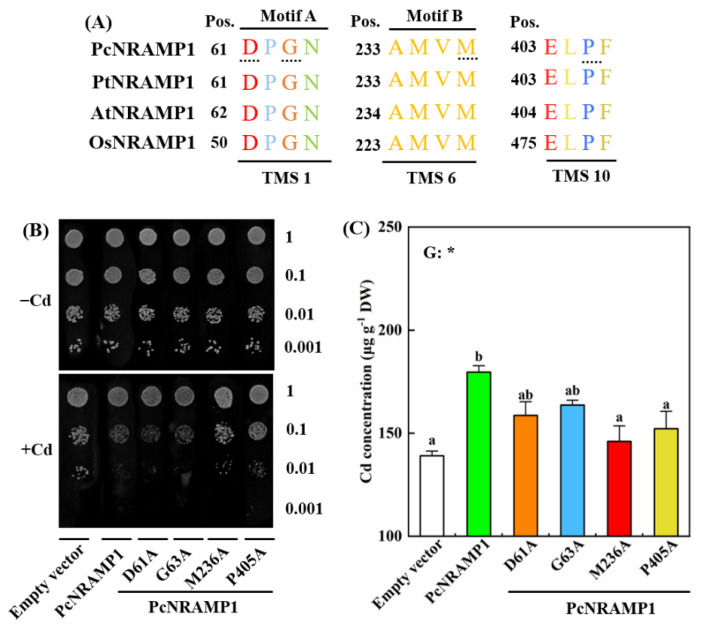
Transport activities of Cd of mutated PcNRAMP1 in yeast. (**A**) Sequence alignment of different transmembrane segments of PcNRAMP1 and its homologous proteins in *P. trichocarpa* (Pt), *A.thaliana* (At), and *O.sativa* (Os). TMS: transmembrane segments. Dashed line in panel (**A**): selected mutation sites. (**B**) The growth status of ∆*ycf1* transformed with empty vector, *PcNRAMP1*, and the mutated *PcNRAMP1* (*PcNRAMP1*^D61A^, *PcNRAMP1*^G63A^, *PcNRAMP1*^M236A^ and *PcNRAMP1*^P405A^) under 0 (−Cd) or 30 μM (+Cd) CdCl_2_ for 4 days. (**C**) Cd concentration in yeast cells transformed with mutated *PcNRAMP1*. Bars indicate means ± SE (n = 4). Different letters on the bars indicate significant differences. The *p*-value of the one-way ANOVA of genotypes (G) is also indicated. *: *p* < 0.05.

To further identify which amino acid residues are critical for Cd transport activity of PcNRAMP1, conserved amino acid residues were selected for the mutation from different TMS of PcNRAMP1, i.e., D61/G63, TMS1; M236, TMS6 and P405, TMS10 (Figure 7A). The Cd-sensitive mutant yeast strain ∆*ycf1* was transformed with pYES2-*PcNRAMP1* plasmids carrying the individual mutations of D61A, G63A, M236A and P405A, pYES2-*PcNRAMP1* and the empty vector (pYES2). Under 0 μM Cd condition, the growth of the yeasts expressing *PcNRAMP1* mutants (*PcNRAMP1*^D61A^, *PcNRAMP1*^G63A^, *PcNRAMP1*^M236A^ and *PcNRAMP1*^P405A^) was similar to that expressing the native *PcNRAMP1* or the empty vector (Figure 7B). Under 30 μM Cd condition, the growth of the yeasts expressing either *PcNRAMP1*^D61A^ or *PcNRAMP1*^G63A^ was similar to that expressing the native *PcNRAMP1* (Figure 7B). Notably, the growth of the yeasts expressing either *PcNRAMP1*^M236A^ or *PcNRAMP1*^P405A^ was better than that expressing the native *PcNRAMP1* on the SG-Ura medium with Cd (Figure 7B). The growth results of these yeasts were further validated by the Cd concentrations in the yeasts expressing *PcNRAMP1* mutations, native *PcNRAMP1* and the empty vector (Figure 7C). These data indicate that the two amino acid residues of PcNRAMP1, i.e., M236 and P405, play a pivotal role in Cd transport activity.

Additionally, the yeast mutant strains ∆*pmr1* and ∆*ccc1* that were hypersensitive to excess Mn and Fe, respectively, were transferred with pYES2-*PcNRAMP1* plasmids carrying the individual mutations of D61A, G63A, M236A and P405A, pYES2-*PcNRAMP1* and the empty vector (pYES2). Under the control condition of Mn or Fe treatments, the growth status was similar regarding the yeasts expressing *PcNRAMP1* mutants (*PcNRAMP1*^D61A^, *PcNRAMP1*^G63A^, *PcNRAMP1*^M236A^ and *PcNRAMP1*^P405A^), *PcNRAMP1* and the empty vector (Figure S3). Under 1 mM Mn condition, the growth of the yeasts expressing either *PcNRAMP1*^G63A^ or *PcNRAMP1*^P405A^ was similar to that expressing the native *PcNRAMP1*, but the growth of the yeasts expressing *PcNRAMP1*^D61A^ or *PcNRAMP1*^M236A^ was better than that expressing the native *PcNRAMP1* (Figure S3). Under 8 mM Fe condition, the growth of the yeasts expressing *PcNRAMP1*^D61A^, *PcNRAMP1*^G63A^ or *PcNRAMP1*^P405A^ was similar to that expressing the native *PcNRAMP1*. However, the growth of the yeast expressing *PcNRAMP1*^M236A^ was better than that expressing the native *PcNRAMP1* (Figure S3). These data imply that the two amino acid residues of PcNRAMP1, i.e., D61 and M236, are critical for Mn transport, and the latter amino acid residue, M236, also plays a role in Fe transport.

## 3. Discussion

### 3.1. PcNRAMP1 Is Involved in Cd Uptake and Accumulation in Poplar

NRAMPs are an evolutionally conserved family of proteins involved in uptake and transport of bivalent HMs, including Cd, Mn and Fe [14]. Although a number of *NRAMP* members have been functionally characterized in some herbaceous plant species [14], limited information is available about the molecular functions of *NRAMPs* involved in absorption and transport of HMs in woody plants [8]. In this study, we identified a total of eight putative *NRAMP* genes from the genome of *P. × canescens*. Intriguingly, nine putative *NRAMP* genes are detected, based on EST database and genomic sequences of *P. trichocarpa*, which has a highly genomic sequence homology with *P. × canescens* [13,35]. Noticeably, two Ethylene Insensitive proteins (EINs) containing an NRAMP-like domain are considered to be NRAMP members in *P. trichocarpa* [13], but were uncounted in the current study, because the EINs are not related with Cd uptake and transport [19]. The number of *NRAMP* members in the poplar genome is slightly more than those in *A. thaliana* (six *NRAMPs*) and *O. sativa* with seven *NRAMPs* [15,20]. The phylogenetic analysis results showed that poplar NRAMP members are divided into two groups, which is similar to the NRAMPs in Arabidopsis and rice, indicating that these two groups of plant NRAMPs could differ in their origins and could have diverged early during evolution [36]. Particularly notable is the fact that the high homology of the amino acid sequence of PcNRAMP1 was identified with those of PtNRAMP1, AtNARMP1, AtNRAMP6 and OsNRAMP3, suggesting that PcNRAMP1 probably plays a pivotal role in the uptake and transport of HMs. *AtNRAMP1* is mainly expressed in the root cells and its product acts as a plasma membrane localized transporter for the uptake of Mn, Fe and Cd [16,19,37]. *AtNRAMP6* is predominantly expressed in the dry seed embryo and, to a lesser extent, in aerial parts, but not in the roots, and the transporter is located at the Golgi/trans-Golgi network involved in regulating the distribution of Fe and Cd between subcellular compartments [17,38]. *OsNRAMP3* is expressed in the node which is a junction of vasculatures connecting leaves, stems and panicles, and its product is a plasma membrane localized transporter, preferentially transporting Mn to young leaves and panicles in low Mn condition [39]. The transcript level of *AtNRAMP1* is induced in Mn-starved roots, but the expression levels of *AtNRAMP6* and *OsNRAMP3* remain unaffected in the roots and shoots with deficiency in Mn, Fe or Zn [16,38,39]. The transcript levels of *NRAMP1* in *Populus* species are induced in response to Cd exposure [34,40]. These results indicate that poplar NRAMP1 is probably involved in Cd uptake and transport.

To further investigate the molecular functions of poplar NRAMP1 involved in Cd transport, we characterized *PcNRAMP1* from *P. × canescens* and found that it is implicated in Cd absorption and transport in poplar plants. We drew this conclusion based on the following evidence: (i) *PcNRAMP1* was predominantly expressed in the roots and its expression was induced by Cd exposure, (ii) PcNRAMP1 was localized to the plasma membrane and displayed Cd transport activity in yeast, and (iii) overexpression of *PcNRAMP1* in *P. × canescens*, not only resulted in growth arrest of poplar plants due to Cd toxicity, but brought about enhanced net Cd^2+^ influxes in poplar roots and greater Cd accumulation in transgenic poplars in comparison with the WT plants.

As transporters, plant *NRAMPs* are often highly expressed in the tissues where they function. Moreover, the expression of *NRAMPs* can be induced in the plants in response to Cd exposure. Currently, AtNRAMP1, AtNRAMP3, AtNRAMP4 and AtNRAMP6 in Arabidopsis, and OsNRAMP1 and OsNRAMP5 in rice, have been characterized as transporters involved in Cd uptake, transport and sequestration [17,19,41,42,43]. In line with *AtNRAMP1* and *OsNRAMP1*, *PcNRAMP1* transcripts were highly expressed in the roots. The transcript levels of *AtNRAMP1* and *OsNRAMP1* increased in the plants exposed to Cd, and the transcriptional expression of *AtNRAMP1* and *OsNRAMP1* was induced by deficiency of manganese (Mn) and iron (Fe), respectively [19,21]. In pak choi (*Arachis hypogaea* L.) and peanut (*Brassica rapa* L.), the mRNA levels of *AhNRAMP1* and *BcNRAMP1* are also up-regulated by Mn or Fe deficiency in the roots [44,45,46]. Interestingly, in this study we found that the expression of *PcNRAMP1* was upregulated in Cd-treated roots, but remained unaltered in response to deficiency of either Mn or Fe. These data indicate that the expression of *PcNRAMP1* in poplars is responsive to Cd exposure and is different in response to deficiency of either Mn or Fe, in comparison with *AtNRAMP1*, *OsNRAMP1*, *AhNRAMP1* and *BcNRAMP1*.

In addition to the transcriptional expression pattern of *PcNRAMP1*, the subcellular localization and Cd transport activity in yeast of PcNRAMP1 demonstrated that this protein is probably involved in Cd uptake in poplars. Similar to the plasma membrane localization of AtNRAMP1 and OsNRAMP1, in this study we found that PcNRAMP1 was localized at the plasma membrane of cells, by using the GFP-PcNRAMP1 fusion protein. Importantly, we found that the expression of *PcNRAMP1* in the Cd-sensitive mutant yeast strain ∆*ycf1* led to increases in Cd sensitivity and Cd accumulation. Previously, it was demonstrated that the growth of wildtype yeast, expressing *AtNRMAP1*, is impaired in comparison with the yeast transformed with the empty vector on a solid medium containing Cd, and greater Cd accumulation was detected in yeast expressing *AtNRAMP1* than in yeast expressing the empty vector in liquid culture [19]. Recently, similar transport activity assays have been carried out in yeasts for several NRAMP1 proteins, including OsNRAMP1, BcNRAMP1 of *Brassica rapa* and MhNRAMP1 of *Malus hupehensis* [41,46,47]. It turns out that these NRAMP1 proteins have the transport activities for Cd when they are expressed in yeasts. Taken together, these results indicate that PcNRAMP1 is a plasma membrane localized protein which is able to transport Cd from a medium into yeast cells.

Despite the fact that PcNRAMP1 was able to absorb Cd in the heterologous yeast assay, we further demonstrated that PcNRAMP1 is a transporter involved in Cd uptake and transport by overexpressing *PcNRAMP1* in *P. × canescens*. Cd in the soil is taken up and transported to the vessels in the stele of the roots through the apoplastic and symplastic pathways before it is translocated to aerial tissues of poplars [9,34]. In the current study, we found higher net Cd^2+^ influxes in the roots, greater Cd accumulation and BCFs of the transgenic poplars compared to those of the WT plants, suggesting more rapid uptake of Cd^2+^ in the roots, and more absorption Cd^2+^ ions, then translocated to the wood, bark and leaf tissues of *P. × canescens*. Consistently, higher Cd^2+^ uptake rates in the roots and greater Cd accumulation in the plants were detected in *NRAMP1*-overexpressed herbaceous plants [46,47,48]. These results imply that PcNRAMP1 is able to take up Cd^2+^ from the soil solution into the root cells and subsequently transports Cd from the roots to the aerial tissues of poplars.

Although *PcNRAMP1*-overexpressed *P. × canescens* accumulated 27.5% more Cd compared to the WT poplar, Cd-induced reductions in the biomass of transgenic poplars were similar to that of the WT plants, suggesting that *P. × canescens* overexpressing *PcNRAMP1* has a greater Cd tolerance in comparison with the WT poplar. The growth performance, such as CO_2_ assimilation rates and plant biomass, was similar between the transgenics and the WT poplars under 0 µM Cd condition, but we found interveinal chlorosis in the mature leaves of *PcNRAMP1*-overexpressed poplars. Since lower concentrations of chlorophylls can lead to foliar chlorosis [49], the interveinal chlorosis in the mature leaves of transgenic poplars was probably associated with the reduced levels of chlorophylls in the transgenics in comparison with that of the WT poplars. Moreover, nutritional imbalance, particularly lower magnesium (Mg) and higher manganese (Mn), can also bring about foliar chlorosis in plants [50]. The interveinal chlorosis was also probably related to lower levels of Mg in the leaves of transgenic poplars compared to the WT plants, which can be ascribed to the increased uptake of Mn and Fe in the roots of transgenic poplars and the translocation of Mn and Fe to aerial tissues. In line with higher levels of Mn and Fe in *PcNRAMP1*-overexpressed poplars in comparison with the WT plants, the expression of *PcNRAMP1* in yeast mutant strains ∆*pmr1* and ∆*ccc1* also enhances the uptake of Mn and Fe from the medium. Higher concentrations of Mn and Fe in *AtNRAMP1*-overexpressed Arabidopsis and lower levels of Mn in the leaves of *osnramp1* mutants have been detected [16,19,21]. These results suggest that PcNRAMP1 is also involved in the uptake and transport of Mn and Fe in *P. × canescens.*

Taken together, our results suggest that PcNRAMP1 is a plasma membrane-localized transporter involved in Cd uptake and transporting Cd from the roots to aerial tissues of *P. × canescens.* Additionally, PcNRAMP1 is probably involved in the uptake of Mn and Fe in the roots, and its activity for Mn uptake, but not for Fe absorption, is inhibited by Cd exposure in the roots of *P. × canescens*.

### 3.2. Conserved Amino Acid Residues in PcNRAMP1 Affect the Uptake Activity of Cd

NRAMPs in plants can not only take up essential nutritional ions, but absorb non-essential metal ions, such as Cd^2+^ [16,46,51], suggesting that NRAMPs generally have poor selectivity for divalent metal ions. The selectivity and the activity of NRAMPs towards divalent transition metal ions are largely determined by the structures of NRAMPs [14,25]. The crystal structures of several NRAMPs from microorganisms and mice have demonstrated that the transmembrane structure and function of the transporter are conservative [25,52,53,54]. TMS of NRAMP proteins play pivotal roles in coupling divalent metal ion transport [55]. Particularly, TMS1 and TMS6 serve as crucial structural segments for metal ion transport [55]. The conserved amino acid residues in TMS of NRAMPs are crucial for the activity and the selectivity of the transporters. For instance, the E401K mutation in TMS10 of AtNRAMP4 deleted its transport activity for Cd^2+^, Zn^2+^ and Mn^2+^, but had no effects on Fe^2+^ uptake [27]. The F413I mutation eliminated Cd^2+^ transport activity and also reduced the transport activity for Zn^2+^ by AtNRAMP4, but hardly affected the transport of Fe^2+^ and Mn^2+^ [27]. Both mutations in TMS1, i.e., L67V and L67I, only impaired the transport of Cd^2+^ by AtNRAMP4 [27]. Moreover, the conserved residues in OsNRAT1 and FeNRAMP5 have been identified and they play essential roles in the selectivity of both transporters for Al and Mn, respectively [28,29]. In line with these findings, several mutations, i.e., D61A, G63A, M236A and P405A in PcNRAMP1, have led to different effects on the growth performance of the Cd-sensitive mutant yeast strain ∆*ycf1* on the Cd-containing medium and on Cd accumulation in the yeast cells. Additionally, the growth inhibition of mutant yeast and Cd accumulation in the yeast cells expressing *PcNRAMP1*^M236A^ and *PcNRAMP1*^P405A^ are similar to those expressing the empty vector, suggesting that both mutations (M236A and P405A) have abolished Cd transport activity of PcNRAMP1. These results suggest that two residues in PcNRAMP1, including M236 and P405, are essential for its Cd transport activity in *P. × canescens*.

**Figure 8:**
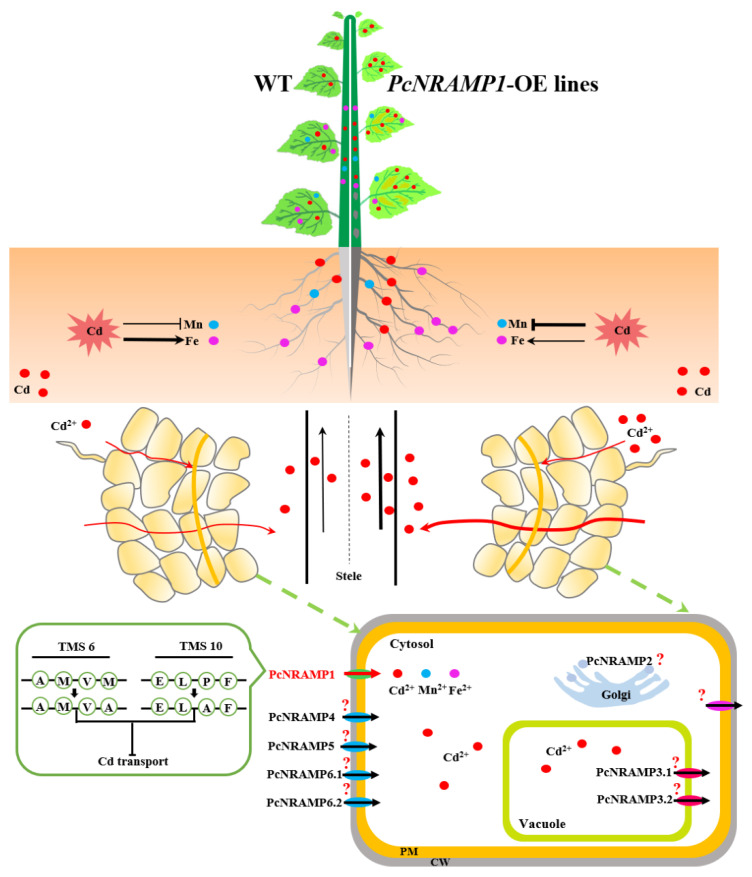
A schematic model illustrating *PcNRAMP* members and the molecular functions of PcNRAMP1 involved in HM uptake and transport of *P. × canescens*. TMS: transmembrane segments, PM: plasma membrane, CW: cell wall. 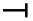: indicating the process is inhibited, 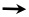: indicating the process is enhanced, and the thickness of both symbols indicates the relative strength of the process.

As summarized in Figure 8, eight putative *NRAMP* members were identified in the poplar genome and most of them were primarily expressed in the roots of *P. × canescens*. The transcriptional expression of *PcNRAMP1* was induced in the roots exposed to Cd and it encoded a plasma membrane-localized protein. PcNRAMP1 demonstrated transport activity for Cd^2+^ when expressed in yeast. *P. × canescens* overexpressing *PcNRAMP1* displayed a similar growth performance to the WT poplars. The transgenic poplars significantly enhanced net Cd^2+^ influxes in the roots and Cd accumulation in the roots and aerial tissues compared to the WT. Cd-induced biomass decreases were similar between the transgenic and WT poplars. The two amino acid residues of PcNRAMP1, i.e., M236 and P405, played pivotal roles in determining transport activity for Cd. These results suggest that PcNRAMP1 is a plasma membrane-localized transporter involved in Cd uptake and transporting Cd from roots to aerial tissues, and the conserved amino acid residues in PcNRAMP1 are essential for Cd transport activity in poplars.

## 4. Materials and Methods

### 4.1. Identification and Phylogenetic Analysis of NRAMP Genes

The NRAMP sequences and genome files of *Populus × canescens*, *P. trichocarpa*, *Arabidopsis thaliana* and *Oryza sativa* were downloaded from the relevant databases (*P. × canescens*: sPta717 Variant DB version 1.1, http://aspendb.uga.edu/index.php/databases/spta-717-genome, accessed on 10 August 2020; *P. trichocarpa*, *A. thaliana* and *O. sativa*: Phytozome version 12.0, https://phytozome.jgi.doe.gov/pz/portal.html, accessed on 10 August 2020). BLASTp and hmmsearch were used to identify the NRAMP proteins in *P. × canescens* according to the method [56]. Briefly, BLASTp searches were conducted in the database of *P. × canescens* (sPta717 Variant DB) using the homologous NRAMP protein sequences of *P. trichocarpa*, *A. thaliana* and *O. sative*. At the same time, the HMMER 3.0 program (https://www.ebi.ac.uk/Tools/hmmer/search/hmmsearch, accessed on 12 August 2020) was employed to identify the NRAMP protein sequences in the genome sequences of *P. × canescens* using the Hidden Markov Model (HMM) of the NRAMP domain file (PF01566). The sequences of candidate PcNRAMPs with NRAMP domain were screened and confirmed by the conserved domains database of the National Center for Biotechnology Information (NCBI) (https://www.ncbi.nlm.nih.gov/cdd, accessed on 18 August 2020), the simple modular architecture research tool (http://smart.embl-heidelberg.de/, accessed on 18 August 2020) and the Pfam database. To investigate the phylogenetic relationships between PcNRAMP proteins and other NRAMP proteins from *P. trichocarpa*, *A. thaliana* and *O. sative*, the phylogenetic tree was constructed using the identified NRAMP protein sequences mentioned above by the neighbor-joining method in MEGA 7.0 (Tokyo Metropolitan University, Hachioji, Japan) with 1000 bootstrap replicates. The phylogenetic tree was visualized by Evolview (www.evolgenius.info/evolview, accessed on 25 August 2020). The molecular weight (MW), theoretical isoelectric point (pI) and grand average of hydropathicity of PcNRAMP proteins were predicted based on the database of ExPASy (https://web.expasy.org/protparam/, accessed on 30 August 2020). The transmembrane helices of PcNRAMP proteins were predicted according to the TMHMM Server (version 2.0, http://www.cbs.dtu.dk/services/TMHMM/, accessed on 30 August 2020).

### 4.2. Plant Growth and Treatments

Plantlets of *P. × canescens* were produced by micropropagation [57] and cultured in a climate chamber (day/night temperature: 25/18 °C, relative air humidity: 50–60%, light per day: 16 h; photosynthetic photon flux: 150 μmol m^−2^ s^−1^). After four weeks, the rooted poplars were cultivated in hydroponics with one-fourth Hoagland nutrient solution. Poplar plants in hydroponics were grown in a greenhouse (day/night temperature: 25/18 °C; relative air humidity: 50–60%). The nutrient solution for hydroponics was renewed every two days.

After cultivation in a greenhouse for two months, the plants of *P. × canescens* (also referred to the wildtype, WT) with similar growth performance were selected and divided into five groups with four plants in each group for the treatments. The plants in two groups were used for Cd treatments and the rest were used for nutrient deficiency treatments. Four poplar plants in the two groups were supplied with either 0 or 100 μM CdCl_2_ for seven days according to a previous study and a preliminary experiment [34]. Four poplars in each of the remaining three groups were supplied with one of the modified one-fourth Hoagland nutrient solutions (no Mn, Fe or Zn for the deficiency in Mn, Fe or Zn, respectively) for seven days.

### 4.3. Gas Exchange Measurement and Harvest

Before harvesting, the CO_2_ assimilation rate (*A*), stomatal conductance (*g_s_*) and transpiration rate (*E*) were measured in three mature leaves of each plant (LPI = 6–8) using a LiCOR-6400 portable photosynthesis system (Li-COR Inc., Lincoln, NE, USA) as suggested [34].

After gas exchange measurements, poplar plants were harvested by separating the root, bark, wood, young and mature leaf tissues. The roots were carefully washed in 50 mM CaCl_2_ for five minutes, and subsequently in sterilized water three times. For each plant, fresh weights of the roots, bark, wood, young and mature leaves were recorded. The samples were immediately frozen in liquid nitrogen, ground into fine powder, and stored at −80 °C for further analysis. To calculate the fresh-to-dry mass ratio, the fresh powder (ca. 100 mg) was dried at 75 °C for 72 h. The biomass was calculated using fresh-to-dry mass ratio multiplied by its fresh weight.

### 4.4. Expression Analysis of PcNRAMPs

Total RNA was isolated from the fresh powder of the root, bark, wood, and young and mature leaf tissues of *P. × canescens* using the CTAB method [58]. Subsequently, the genomic DNA in total RNA samples was removed by DNase treatment and the purified RNA was reverse-transcribed to cDNA using PrimeScript™ RT reagent Kit (RR047A, Takara, Dalian, China) according to the manufacturer’s protocol. The quantitative PCR was performed in a 20 μL reaction using 10 μL 2× TB Green Premix Ex Taq™ II (RR420A, Takara, Dalian, China), 1 μL cDNA, 0.5 μL of 10 μM primers and 8 μL of H_2_O in a real time system (LightCycler^®^ 480 Ⅱ, RoChe, Rotkreuz, Switzerland). Four biological replicates, each with three technical replicates, were carried out. *PcActin2/7* and *PcEF1a* were used as internal reference genes. The relative expression levels of *PcNRAMPs* were calculated using the 2^−^^△△CT^ method [59]. The primers for *PcNRAMPs* and internal reference genes were presented in Table S1.

### 4.5. Cloning the Full-Length cDNA of PcNRAMP1

Total RNA from the roots of *P. × canescens* was isolated and purified using a plant RNA kit (R6827, Omega Bio-Tek, Georgia, USA) as suggested [60]. Subsequently, cDNA was synthesized using Primescript™ 1st strand cDNA synthesis kit (6210A, Takara, Dalian, China) according to the manufacturer’s protocol. The full-length cDNA of *PcNRAMP1* was amplified by specific primers (Table S1). The reaction system of PCR consisted of 50 µL, including 1 μL of cDNA, 0.5 μL of each of the primer (10 μM), 25 μL of Premix Taq™ (RR902A, Takara, Dalian, China) and 23 μL of H_2_O. The PCR was performed at 95 °C for 3 min, followed at 95 °C for 30 s, 55 °C for 30 s, and 72 °C for 1 min 30 s with 31 cycles, and extended at 72 °C for 5 min. The PCR product was purified by gel extraction kit (DP209, Tiangen Biotech Co., Ltd. Beijing, China) and cloned into the pMD19-T vector for sequencing.

### 4.6. Determination of PcNRAMP1 Subcellular Localization

The full-length cDNA of *PcNRAMP1* was amplified and ligated into Gateway entry vector pDONR222 (Invitrogen™, Shanghai, China). After sequencing, the cDNA was directionally cloned into pK7WGF to generate *PcNRAMP1*-pK7WGF, in which *PcNRAMP1* was fused to the C-terminal of EGFP under the control of the CaMV35S promoter (*GFP-PcNRAMP1*) as described elsewhere [5]. The primer sequences used were listed in Table S1. Then, GFP-PcNRAMP1 was co-transformed with pMDC32-1A CAN2b-mCherry (a plasma membrane marker) into *Nicotiana benthamiana* leaf epidermal cells by agroinfiltration [5]. After infiltration for 48 h, the subcellular localization of PcNRAMP1 was detected by a confocal laser scanning microscope (LSM880, Carl Zeiss, Jena, Germany). The excitation/emission wavelengths were 488 nm/510 to 530 nm for green fluorescence, and 587 nm/610 nm for red fluorescence.

### 4.7. Yeast Expression Assay

*Saccharomyces cerevisiae* yeast mutant strain Δ*ycf1* (Cd-hypersensitive) was used for the yeast expression assay of *PcNRAMP1* according to the method in [5]. The coding sequence of *PcNRAMP1* was recombination-cloned into pYES2 vector (Invitrogen™ 12286019) and named as *PcNRAMP1-pYES2*. *PcNRAMP1-pYES2* plasmids and the empty pYES2 vector were expressed in yeast mutant strain Δ*ycf1* as described [61]. For the Cd sensitivity assay, transformed yeast cells were cultured in a liquid synthetic defined medium (SD-Ura) with 2% glucose to their optical density (OD_600_ = 1.0). The yeast cells were diluted to optical densities at 600 nm of 1, 0.1, 0.01, and 0.001. Then, the diluted yeast cells were spotted on the plates containing SD-Ura solid medium supplied with 2% galactose/glucose in combination with either 0 (−Cd) or 30 μM Cd (+Cd). The plates were incubated for four days at 30 °C and photographed.

To construct the yeast growth curves, pre-cultured transformed yeast cells (100 μL, OD_600_ = 1.0) were added to liquid medium (SD-Ura, 2% galactose) containing 30 μM Cd based on the method with minor modifications [5]. The OD_600_ values of yeast cells were recorded at 0, 12, 24, 36, 48, 60 and 72 h. Afterwards, the yeast cells in the liquid medium were centrifugated. The pellets were washed using 50 mM CaCl_2_ once and further washed using sterilized water three times. The pellets were incubated at 80 °C for five days, and the dry weight of the pellets was recorded.

### 4.8. Transformation of P. × canescens and Cd Exposure

The full length of *PcNRAMP1* was directionally cloned into the pK2GW7 binary overexpression vector [62]. The recombinant plasmid was named as pK2GW7*-PcNRAMP1* and the empty vector (pK2GW7) was introduced into the *Agrobacterium tumefaciens*. The success of the transformation was confirmed by PCR. The transgenic plants of *P. × canescens* were produced according to the transformation protocol in [63]. Briefly, the stems of *P. × canescens* plants grown in a climate chamber for six weeks were cut into segments (without nodes) as explants. These explants were incubated in *Agrobacterium* culture (A*grobacterium tumefaciens* strain GV3101 containing either pK2GW7-*PcNRAMP1* or the empty vector pK2GW7, OD = 0.5) for 30 min at 28 °C. Subsequently, the explants with *Agrobacterium* were co-cultivated on Petri dishes containing half-strength MS medium (2% sucrose) for two to three days in the dark. Then, the explants were transferred to Petri dishes with half-strength MS medium containing 2% sucrose, 50 mg L^−1^ kanamycin, 150 mg L^−1^ cefotaxime, 200 mg L^−1^ ticarcillin clavulanate and 0.0022 mg L^−1^ thidiazuron. After ca. six weeks, the explants with new sprouts were transferred to new Petri dishes with the same medium for further growth under low light. The new shoots formed from the explants were cut and transferred to the medium mentioned above without thidiazuron for rooting. The transformed *PcNRAMP1* or the empty vector in the rooted cuttings were verified by PCR. The transgenic lines with high expression levels of *PcNRAMP1* were selected for further experiments using RT-PCR, and *PcActin* was used as a reference gene.

Plantlets of wildtype (WT, containing the empty vector) and transgenic *P. × canescens* were micropropagated and cultured in a climate chamber as mentioned above. After four weeks, the rooted poplars were transferred to the hydroponics supplied with one-fourth Hoagland solution in the greenhouse. After two months, WT and three transgenic lines of poplars were divided into four groups. Each group was further divided into two subgroups (each subgroup with four plants). The plants in each subgroup were treated with either 0 or 100 μM CdCl_2_ for two weeks. Afterwards, the plants were harvested as mentioned above. Notably, the photosynthesis in the mature leaves (see above) and the net Cd^2+^ fluxes in the fine roots (see below) were determined before harvesting.

### 4.9. Detection of Net Cd^2+^ Fluxes

The net Cd^2+^ flux in the fine roots was determined by the non-invasive micro-test technique (NMT-YG-100, Younger USA LLC, Amherst, MA, USA) as described previously [34]. Briefly, the fine roots excised from the plants exposed to either 0 (−Cd) or 100 (+Cd) μM CdCl_2_ for two weeks were immediately transferred to a Petri dish containing 10 mL of measuring solution (0.1 mM CdCl_2_, 0.5 mM KCl, 0.1 mM MgCl_2_ and 0.05 mM CaCl_2_, pH 5.8). The net Cd^2+^ fluxes were detected in four white fine roots from each plant of WT and transgenic lines treated with either 0 or 100 μM CdCl_2_. Four plants for each treatment were analyzed.

In order to determine the positions where the maximal Cd^2+^ flux occurred along the roots of WT and transgenic lines exposed to 0 or 100 μM CdCl_2_, a preliminary experiment was carried out by taking measurements at the root apex at 300 µm interval in the region from 0 to 2100 µm along the root tip (Figure S1A). The Cd^2+^ gradients near to the root surface (ca. 2–5 µm) were measured by moving the Cd^2+^-selective microelectrode between two positions (at a distance of 30 µm) perpendicular to the root axis.

### 4.10. Determination of Metal Concentration, Cd Amounts, Cd Bio-Concentration Factor (BCF), and Chlorophyll Concentration

The concentrations of Cd, Mn, Fe, Zn, Ca and Mg in the root, wood, bark and leaf tissues, and also Cd in yeast mycelia were determined using an ICP-MS according to the method in [60]. The total Cd amount per plant was the sum of Cd amount in each tissue, which was calculated by multiplying the biomass of each tissue (root, wood, bark and leaves) with the Cd concentration in the corresponding tissue [34]. The bio-concentration factor (BCF) in the WT and transgenic lines were calculated as described elsewhere [34]. The chlorophyll concentrations in the leaves were analyzed as proposed in [34].

### 4.11. Mutagenesis and Yeast Selection

The mutagenesis sites were selected based on the conservation analysis of amino acid residues in different transmembrane segments of PcNRAMP1 and its homologues in *P. trichocarpa*, *A. thaliana* and *O. sativa*. The mutagenesis in *PcNRAMP1* was carried out as described by Pottier et al. [27]. Briefly, site-directed mutation was performed by changing motif A of TMS1 from DPGN to APGN or DPAN using a Fast Site-Directed Mutagenesis Kit (KM101, Tiangen Biotech Co., Ltd. Beijing, China). Similarly, site-directed mutation in TMS6 (AMVM→AMVA) and TMS 10 (ELPF→ELAF) was performed. The primer sequences used were listed in Table S1. *PcNRAMP1* mutants, *PcNRAMP1* and empty vector plasmids were transformed to yeast mutant strain Δ*ycf1* as described above. The Cd sensitivity assay and Cd concentration in the yeast cells were determined as described above. Briefly, the yeast cells transferred with mutated *PcNRAMP1*, native *PcNRAMP1* or empty vector were cultured in a liquid defined medium (SD-Ura with glucose, in which PcNRAMP1 protein was unable to be expressed) to their optical density (OD_600_ = 1.0). Subsequently, one milliliter of the solution containing the yeast cells was transferred to the other defined liquid medium (SG-Ura with galactose, where PcNRAMP1 protein can be expressed) and cultured. Finally, these cultured yeast cells were harvested and used for the measurement of Cd concentration.

To examine the role of PcNRAMP1 in transporting the ions of manganese (Mn) and iron (Fe), yeast mutant strains ∆*pmr1* and ∆*ccc1* hypersensitive to excess Mn and Fe, respectively, were transformed with the empty vector (pYES2), *PcNRAMP1* or the mutated *PcNRAMP1* (*PcNRAMP1*^D61A^, *PcNRAMP1*^G63A^, *PcNRAMP1*^M236A^ and *PcNRAMP1*^P405A^). The yeast cells diluted with OD_600_ nm of 1–0.001 were cultured in the plate with galactose under either control (Con) or the bivalent metals including 1 (+Mn) mM MnSO_4_ and 8 (+Fe) mM FeSO_4_ for 4 days.

### 4.12. Statistical Analysis

Statistical analysis was performed using Statgraphics Centurion XVI.I (STN, St. Louis, MO, USA). One-way ANOVA (metal treatments or genotypes as a factor) was applied to the analyses of the expression levels of *PcNRAMPs* in the roots of *P. × canescens* and of the Cd concentrations in yeast cells. Two-way ANOVA tests (Cd and genotype (G) as the two main factors) were applied to the analyses of net Cd^2+^ fluxes, mean fluxes of Cd^2+^, Cd concentrations and amounts, BCF and photosynthetic parameters. The data were tested for normality before statistical analyses. Differences between the means were considered to be significant when the *p* ≤ 0.05, based on the ANOVA F-test. Posteriori comparisons of the means were performed using LSD’s method. The gene expression heatmap in different tissues was generated using TBtools (South China Agricultural University, Guangzhou, China) [64]. Net Cd^2+^ fluxes were calculated using the program JCal V3.2.1 (Xuyue (Beijing) Sci and Tech Co. Ltd., Beijing, China) as described [34].

## Data Availability

Data is contained within the article or Supplementary Material.

## Supplementary Materials

The following supporting information can be downloaded at: https://www.mdpi.com/article/10.3390/ijms23147593/s1.
