# Supplementary material for: PcNRAMP1 Enhances Cadmium Uptake and Accumulation in Populus × canescens
Source: Int J Mol Sci. 2022 Jul 8;23(14):7593. doi: 10.3390/ijms23147593 (PMC9316961; doi:10.3390/ijms23147593)
Supplement: Supplementary file 1 [file ijms-23-07593-s001.zip › ijms-1772605-supplementary.pdf]

Table S1. Primer sequences used in this study

Name	Forward (5'- 3')	Reverse (5'- 3')	Applications
PcNRAMP1	**TGAAAATCCGCAAATCGC**	**TCCACAAAGATGTTCGGTCAT**	
PcNRAMP2	**AGGATTATTCCGATGAAGTAGC**	**GTTGCCCACAGTAAGAGCC**	
PcNRAMP3.1	**CTCCTGGTGCTGTCAATCTAA**	**GGCACGCCTGGAAAGAT**	
PcNRAMP3.2	**AGCTTTTCAAACTAAACGGGAG**	**TCTGATGTCATCGCTTAGCAA**	
PcNRAMP4	**TGGGAATTATTGGCATCAACA**	**TCTGAACACCCTCTATTATGGC**	
PcNRAMP5	**GCCCACTTGTCGCTTTACT**	**GTGGATTGACAACCCGAAG**	**RT-qPCR**
PcNRAMP6.1	**ATGGCAATCTATATCCTAGCAG**	**CAAGTCCGTTTTCCATGTTA**	
PcNRAMP6.2	**CACCACTATCAAATCGAGGTT**	**TTTTCAAGGCAATAGCAAT**	
PcActin2/7	**CCCATTGAGCACGGTATTGT**	**TACGACCACTGGCATACAGG**	
PcEF1α	**TCCCTTTGTCCCAATCTCTG**	**ACATCCTGAAGTGGGAGACG**	
PcNRAMP1	**ATGGCGGGTTCGTCTTCTGT**	**CAAATCATTTGCATTACTCCTC**	**Clone**
**GW-*PcNRAMP1***	**GGGGACAAGTTTGTACAAAAAAGCAGGCTTAATGGC GGGTTCGTCTTCTGT**	**GGGGACCACTTTGTACAAGAAAGCTGGGTCTTACAA ATCATTTGCATTACTC**	**Gateway**

Table S1. Continued

Name	Forward (5'- 3')	Reverse (5'- 3')	Applications
35S-PcNRAMP1	TCCATTGCCCAGCTATCTGTCAC	ACTCATACTTGTATTGCGCTCCT	
***PcNRAMP1*-RT**	GCACCATTGATTGAGAATTCAGA	TCATACTTGTATTGCGCTCCT	The selection of transgenic lines
PcActin	TCATCGGAATGGAAGCTGCTGGTA	TCATCGGAATGGAAGCTGCTGGTA	
PcNRAMP1^{D61A}	TTGTTTCAATTGCATACATTGCCCCTGG	GCAATGTATGCAATTGAAACAAGGAACCCGGGA	
PcNRAMP1^{G63A}	GCATACATTGACCCTGCAAATTTTGAAACTGACCT GC	GCAGGGTCAATGTATGCAATTGAAACAAGGA	
PcNRAMP1^{M236A}	CACTTCTCGGTGCCATGGTTGCGCCGCACAATCTC CATTC	GAATGGAGATTGTGCGGCGCAACCATGGCACCGAGA AGTG	Site-directed mutations
PcNRAMP1^{P405A}	CGATGATTCTATCTTTTGAGCTCGCTTTTGCTCTCG	CGAGCTCAAAAGATAGAATCATCGATGCAATAATG	

Table S2. Accession numbers of *NRAMP* genes presented in Figure. 1.

Gene Name	Locus names or Accession nos. or AGI codes	Species
AtNRAMP1	AT1G80830	*Arabidopsis thaliana*
AtNRAMP2	AT1G47240	*A. thaliana*
AtNRAMP3	AT2G23150	*A. thaliana*
AtNRAMP4	AT5G67330	*A. thaliana*
AtNRAMP5	AT4G18790	*A. thaliana*
AtNRAMP6	AT1G15960	*A. thaliana*
OsNRAMP1	Os07g0258400	*Oryza sativa*
OsNRAMP2	Os03g0208500	*O. sativa*
OsNRAMP3	Os06g0676000	*O. sativa*
OsNRAT1	Os02g0131800	*O. sativa*
OsNRAMP5	Os07g0257200	*O. sativa*
OsNRAMP6	Os01g0503400	*O. sativa*
OsNRAMP7	Os12g0581600	*O. sativa*
PcNRAMP1	Spta717-Potri.001G044900	*Populus × canescens*
PcNRAMP2	Spta717-Potri.002G121000	*P. × canescens*
PcNRAMP3.1	Spta717-Potri.007G050600	*P. × canescens*
PcNRAMP3.2	Spta717-Potri.007G050700	*P. × canescens*
PcNRAMP4	Spta717-Potri.002G080400	*P. × canescens*
PcNRAMP5	Spta717-Potri.002G080500	*P. × canescens*
PcNRAMP6.1	Spta717-Potri.005G181000	*P. × canescens*
PcNRAMP6.2	Spta717-Potri.005G181100	*P. × canescens*
PtNRAMP1	Potri.001G044900	*Populus trichocarpa*
PtNRAMP2	Potri.002G121000	*P. trichocarpa*
PtNRAMP3.1	Potri.007G050600	*P. trichocarpa*
PtNRAMP3.2	Potri.007G050700	*P. trichocarpa*

Table S2. Continued

Gene Name	Locus names or Accession nos. or AGI codes	*Species*
PtNRAMP4	**Potri.002G080400**	***P. trichocarpa***
PtNRAMP5	**Potri.002G080500**	***P. trichocarpa***
PtNRAMP6.1	**Potri.005G181000**	***P. trichocarpa***
PtNRAMP6.2	**Potri.005G181100**	***P. trichocarpa***

Table S3. Characteristics of the sequences of *PcNRAMP* genes and their proteins

Gene name	Accession number	Chromosomal location	Length of CDS (bp)	Number of amino acids (aa)	Molecular weight (KDa)	Theoretical pI	Grand average of hydropathicity	Transmembrane helices
PcNRAMP1	**Potri.001G044900**	**1**	**1626**	**541**	**58.65**	**8.6**	**0.571**	**12**
PcNRAMP2	**Potri.002G121000**	**2**	**1611**	**536**	**58.61**	**5.19**	**0.496**	**11**
PcNRAMP3.1	**Potri.007G050600**	**7**	**1518**	**505**	**55.51**	**5.01**	**0.65**	**11**
PcNRAMP3.2	**Potri.007G050700**	**7**	**1503**	**500**	**54.64**	**4.93**	**0.667**	**11**
PcNRAMP4	**Potri.002G080400**	**2**	**1659**	**552**	**60.1**	**8.23**	**0.442**	**12**
PcNRAMP5	**Potri.002G080500**	**2**	**1641**	**546**	**58.96**	**7.06**	**0.548**	**12**
PcNRAMP6.1	**Potri.005G181000**	**5**	**1746**	**581**	**63.4**	**7.56**	**0.512**	**12**
PcNRAMP6.2	**Potri.005G181100**	**5**	**1758**	**585**	**63.95**	**8.42**	**0.463**	**10**

Table S4. Biomass of WT and transgenic lines (*PcNRAMP1*-OE5, *PcNRAMP1*-OE8, *PcNRAMP1*-OE9) of *P.* × *canescens* treated with either 0 (−Cd) or 100 (+Cd) μM $CdCl_2$ for 2 weeks. Data indicate means ± SE (n = 4). Different letters after the values indicate significant differences between the treatments. *P*-values of the two-way ANOVAs of genotype (G), Cd and their interactions (G × Cd) are also indicated. *: *P*<0.05; **: *P*<0.01; ***: *P*<0.001; ****: *P*<0.0001; ns: not significant.

Genotype	Cd (μM)	Root	Wood	Bark	Leaf	Whole plant
WT	0	0.64 ± 0.08 b	1.83 ± 0.48 c	1.38 ± 0.10 a	7.50 ± 0.29 a	11.34 ± 0.73 a
	100	0.31 ± 0.01a	1.00 ± 0.13 ab	0.72 ± 0.01 b	5.62 ± 0.27 b	7.69 ± 0.34 b
PcNRAMP1-OE5	0	0.58 ± 0.17 ab	1.97 ± 0.48 c	1.34 ± 0.12 a	7.58 ± 1.2 a	11.47 ±1.74 a
	100	0.37 ± 0.02 ab	0.84 ± 0.04 a	0.73 ± 0.07 b	5.42 ± 0.16 b	7.34 ± 0.06 b
PcNRAMP1-OE8	0	0.53 ± 0.03 ab	1.71 ± 0.06 bc	1.50 ± 0.20 a	7.31 ± 0.62 a	11.05 ± 0.51 a
	100	0.33 ± 0.03 a	0.95 ± 0.05 ab	0.61 ± 0.04 b	5.26 ± 0.26 b	7.15 ± 0.21 b
PcNRAMP1-OE9	0	0.60 ± 0.14 b	2.26 ± 0.22 c	1.40 ± 0.48 a	7.42 ± 0.23 a	11.80 ± 0.66 a
	100	0.33 ± 0.02 a	0.77 ± 0.08 a	0.66 ± 0.06 b	5.33 ± 0.28 b	7.08 ± 0.40 b
P-values	G	ns	ns	ns	ns	ns
	Cd	**	****	****	****	****
	G x Cd	ns	ns	ns	ns	ns

Table S5. Concentrations of Mn, Fe, Zn, Ca and Mg in different tissues of WT and transgenic lines (*PcNRAMP1*-OE5, *PcNRAMP1*-OE8, *PcNRAMP1*-OE9) of *P.* × *canescens* treated with either 0 (−Cd) or 100 (+Cd) μM $CdCl_2$ for 2 weeks. Data indicate means ± SE (n = 4). Different letters after the values indicate significant differences between the treatments. *P*-values of the two-way ANOVAs of genotype (G), Cd and their interactions (G × Cd) are also indicated. *: *P*<0.05; **: *P*<0.01; ***: *P*<0.001; ****: *P*<0.0001; ns: not significant.

Genotype	Cd (μM)	Root					Wood				
		Mn ($\mu g\ g^{-1}$ DW)	Fe ($\mu g\ g^{-1}$ DW)	Zn ($\mu g\ g^{-1}$ DW)	Ca ($mg\ g^{-1}$ DW)	Mg ($mg\ g^{-1}$ DW)	Mn ($\mu g\ g^{-1}$ DW)	Fe ($\mu g\ g^{-1}$ DW)	Zn ($\mu g\ g^{-1}$ DW)	Ca ($mg\ g^{-1}$ DW)	Mg ($mg\ g^{-1}$ DW)
WT	0	14.11 ± 1.94 a	275.80 ± 48.88 a	30.87 ± 4.03 ab	1.75 ± 0.53 a	6.50 ± 0.28 c	2.18 ± 0.22 a	152.73 ± 17.52 a	12.60 ± 0.94 a	0.75 ± 0.01 ab	0.46 ± 0.06 a
	100	11.28 ± 4.09 a	570.98 ± 31.70 b	48.05 ± 5.02 cd	4.20 ± 0.14 c	1.03 ± 0.08 ab	0.46 ± 0.07 a	136.65 ± 3.52 a	13.69 ± 3.08 ab	0.65 ± 0.01 a	0.36 ± 0.06 a
PcNRAMP1-OE5	0	156.19 ± 9.49 b	691.88 ± 117.55 b	40.63 ± 3.29 bc	1.62 ± 0.11 a	0.70 ± 0.05 a	47.62 ± 12.58 b	338.37 ± 18.50 b	18.42 ± 1.36 cd	0.97 ± 0.03 c	0.33 ± 0.03 a
	100	9.62 ± 0.89 ab	1041.06 ± 61.22 c	27.00 ± 2.98 a	3.09 ± 0.16 b	0.71 ± 0.01 a	1.92 ± 0.2 a	126.93 ± 9.01 a	14.36 ± 0.94 ab	0.87 ± 0.04 bc	0.42 ± 0.08 a
PcNRAMP1-OE8	0	183.90 ± 8.66 bc	708.94 ± 33.50 b	48.62 ± 0.7 cd	1.62 ± 0.05 a	0.94 ± 0.03 ab	46.24 ± 0.99 b	298.96 ± 19.18 b	21.75 ± 0.50 de	1.16 ± 0.05 d	0.34 ± 0.03 a
	100	4.24 ± 1.19 a	927.35 ± 18.83 c	37.5 ± 0.47 ab	3.23 ± 0.16 b	0.73 ± 0.02 a	0.8 ± 0.14 a	124.11 ± 0.23 a	18.24 ± 0.78 bc	0.91 ± 0.01 c	0.44 ± 0.05 a
PcNRAMP1-OE9	0	171.68 ± 7.65 c	713.26 ± 61.33 b	56.61 ± 2.18 d	1.62 ± 0.18 a	1.17 ± 0.01 b	58.01 ± 2.62 b	303.47 ± 8.10 b	24.95 ± 1.35 e	1.36 ± 0.12 e	0.35 ± 0.04 a
	100	9.80 ± 0.98 ab	1106.96 ±71.60 c	47.99 ± 3.63 cd	3.37 ± 0.18 b	0.74 ± 0.04 a	1.16 ± 0.4 a	124.89 ± 1.97 a	19.88 ± 0.61 d	0.96 ± 0.03 c	0.47 ± 0.02 a
P-values	G	****	****	**	*	**	****	****	****	****	ns
	Cd	****	****	ns	****	***	****	****	*	****	ns
	G × Cd	****	ns	**	ns	**	****	****	ns	*	ns

Table S5. Continued

Genotype	Cd (μM)	Bark Mn (μg g^{-1} DW)	Bark Fe (μg g^{-1} DW)	Bark Zn (μg g^{-1} DW)	Bark Ca (mg g^{-1} DW)	Bark Mg (mg g^{-1} DW)	Leaf Mn (μg g^{-1} DW)	Leaf Fe (μg g^{-1} DW)	Leaf Zn (μg g^{-1} DW)	Leaf Ca (mg g^{-1} DW)	Leaf Mg (mg g^{-1} DW)
**WT**	**0**	**13.8 ± 1.56 a**	**140.21 ± 9.05 a**	**136.48 ± 16.39 a**	**12.85 ± 1.57 b**	**2.73 ± 0.55 b**	**28.21 ± 1.77 b**	**240.54 ± 3.77 bc**	**53.24 ± 1.20 ab**	**7.04 ± 0.96 d**	**3.48 ± 0.43 d**
	**100**	**3.75 ± 0.15 a**	**108.72 ± 7.92 a**	**114.49 ± 11.36 a**	**6.82 ± 0.169 a**	**2.37 ± 0.52 ab**	**7.97 ± 0.91 a**	**228.60 ± 9.76 b**	**37.49 ± 2.58 a**	**2.60 ± 0.04 a**	**1.42 ± 0.26 ab**
***PcNRAMP1*-OE5**	**0**	**332.66 ± 36.57 b**	**775.91 ± 31.92 b**	**155.03 ± 11.01 ab**	**11.84 ± 0.92 b**	**1.78 ± 0.03 ab**	**51.91 ± 12.71 c**	**283.50 ± 24.19 cd**	**71.94 ± 9.51 cd**	**7.91 ± 0.74 d**	**2.25 ± 0.35 c**
	**100**	**16.58 ± 1.71 a**	**155.24 ± 0.09 a**	**121.34 ± 16.22 a**	**7.75 ± 0.34 a**	**1.51 ± 0.16 a**	**21.15 ± 1.57 ab**	**171.94 ± 20.98 a**	**40.74 ± 4.42 a**	**3.56 ± 0.07 ab**	**1.26 ± 0.01 a**
***PcNRAMP1*-OE8**	**0**	**337.68 ± 6.96 b**	**778.16 ± 43.45 b**	**193.79 ± 7.05 bc**	**11.87 ± 0.48 b**	**1.75 ± 0.04 ab**	**54.03 ± 7.15 c**	**298.65 ± 4.06 d**	**82.7 ± 4.02 de**	**7.38 ± 0.65 d**	**2.13 ± 0.24 bc**
	**100**	**7.94 ± 1.94 a**	**145.22 ± 5.73 a**	**120.39 ± 10.85 a**	**7.93 ± 0.06 a**	**1.46 ± 0.13 a**	**12.35 ± 1.27 a**	**171.62 ± 13.36 a**	**66.7 ± 2.23 bc**	**4.22 ± 0.19 ab**	**1.27 ± 0.02 a**
***PcNRAMP1*-OE9**	**0**	**359.33 ± 20.97 b**	**715.89 ± 19.65 b**	**226.96 ± 11.50 c**	**11.90 ± 0.15 b**	**1.72 ± 0.05 ab**	**51.38 ± 5.22 c**	**308.29 ± 18.45 d**	**93.45 ± 6.70 e**	**6.86 ± 1.32 cd**	**2.01 ± 0.20 b**
	**100**	**9.35 ± 0.45 a**	**150.20 ± 9.51 a**	**119.45 ± 6.72 a**	**8.45 ± 0.30 a**	**1.41 ± 0.15 a**	**14.31 ± 0.57 ab**	**180.62 ± 21.12 a**	**85.69 ± 8.68 de**	**4.88 ± 0.40 bc**	**1.28 ± 0.06 a**
***P*-values**	**G**	****	****	**	ns	*	*	ns	****	ns	*
	**Cd**	****	****	****	****	ns	****	****	***	****	****
	**G × Cd**	****	****	**	ns	ns	ns	**	ns	ns	ns

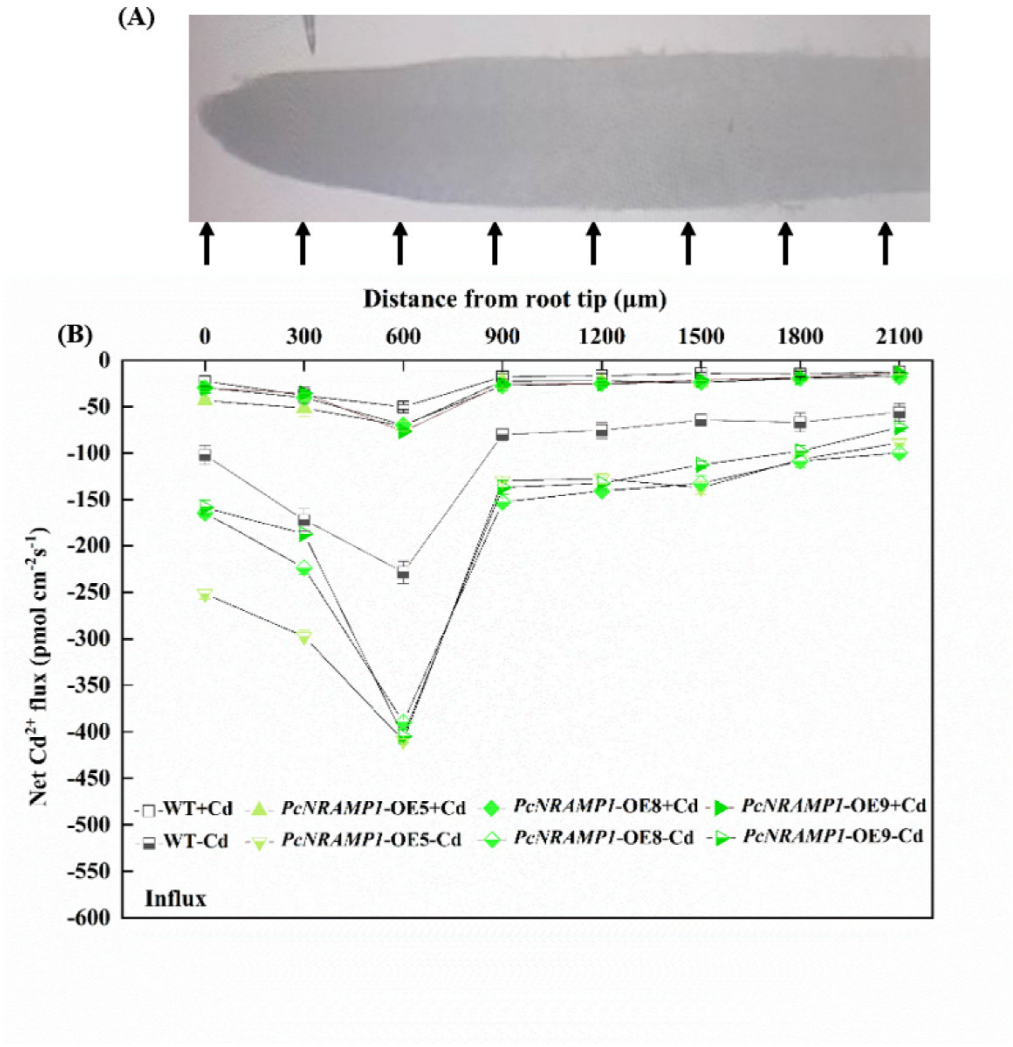

Figure S1. Root tip and net Cd^{2+} fluxes along root apexes. (A-B) Root tip and net Cd^{2+} fluxes along root apexes of WT and transgenic lines (*PcNRAMP1*-OE5, *PcNRAMP1*-OE8, *PcNRAMP1*-OE9) of *P.* × *canescens* exposed to either 0 (−Cd) or 100 (+Cd) μM $CdCl_2$ for 2 weeks. Data indicate means ± SE (n = 4).

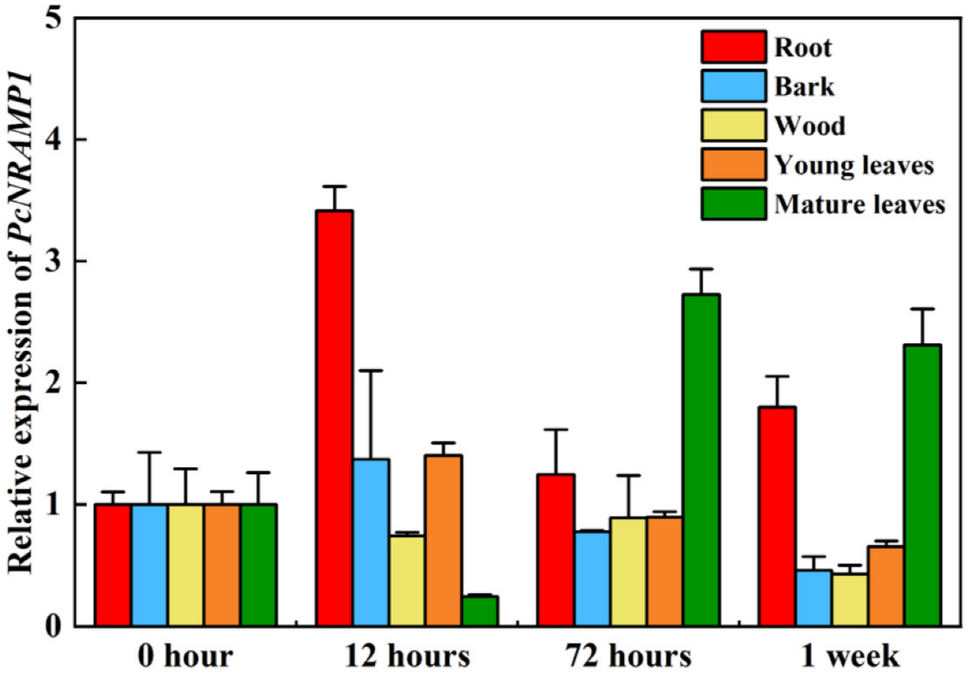

Figure S2. The expression levels of *PcNRAMP1* in different tissues of *P.* × *canescens* exposed to Cd for different times. Bars indicate means ± SE (n = 4). The expression level of *PcNRAMP1* was set to 1 in different tissues at the beginning of the treatment (0 hour), and the corresponding fold changes were calculated at the other time points.

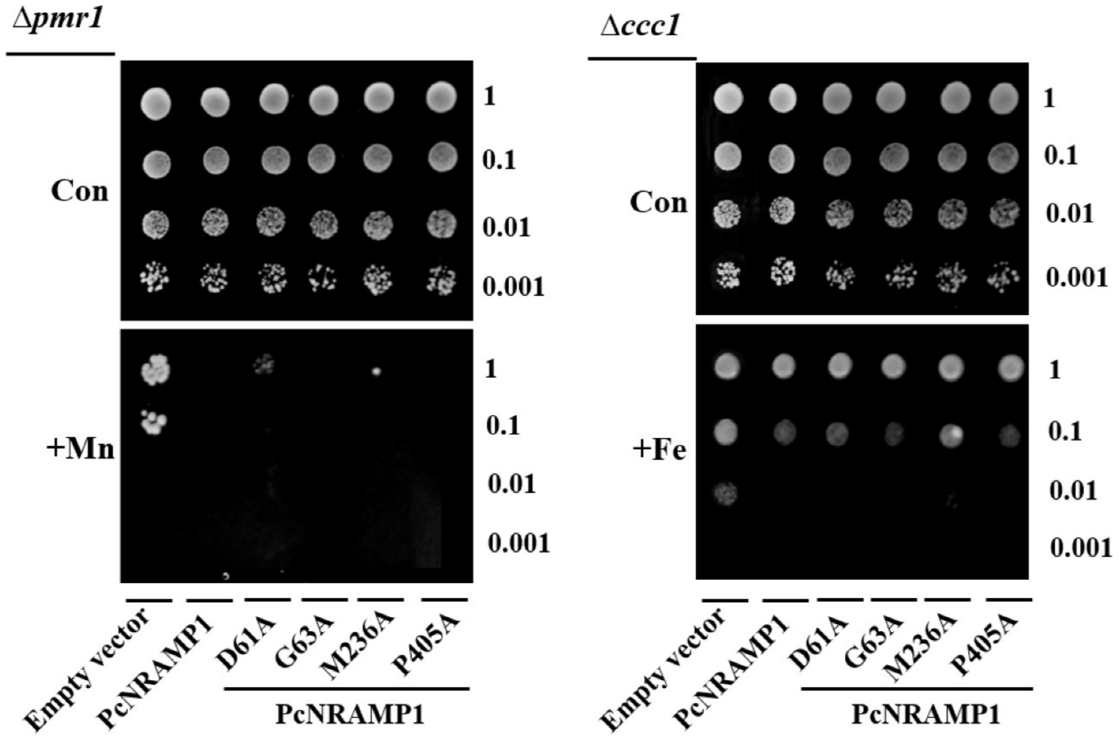

Figure S3. The growth status of yeast mutant strains Δ*pmr1* and Δ*ccc1* cells transformed with empty vector (pYES2), *PcNRAMP1*, or the mutated *PcNRAMP1*. The growth status of yeast mutant strains Δ*pmr1* and Δ*ccc1* hypersensitive to excess Mn and Fe, respectively, cells in which empty vector (pYES2), *PcNRAMP1*, or the mutated *PcNRAMP1* (*PcNRAMP1*^{D61A}, *PcNRAMP1*^{G63A}, *PcNRAMP1*^{M236A} and *PcNRAMP1*^{P405A}) was transformed. The yeast cells diluted with OD600 nm of 1-0.001 were cultured in the plate with galactose under either control (Con) or other bivalent metals including 1 (+Mn) mM $MnSO_4$ and 8 (+Fe) mM $FeSO_4$ for 4 days.

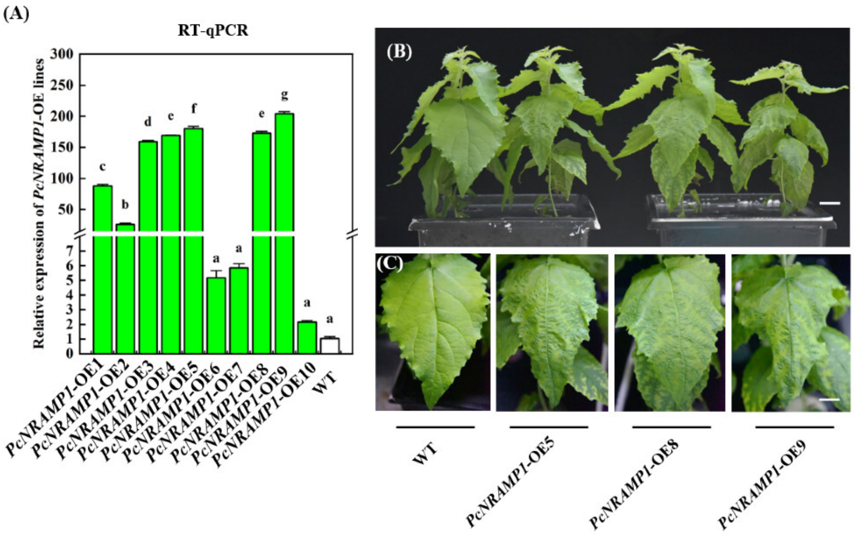

Figure S4. The selection of transgenic lines and the phenotypes of WT and poplars overexpressing *PcNRAMP1*. (A) The selection of transgenic lines. (B-C) The phenotypes of the aboveground plant and mature leaves (LPI=8) of WT and *PcNRAMP1*-overexpressed *P.* × *canescens* supplied with one-fourth Hoagland solution for 1 month. Scale bars in panels B and C indicate 5 and 2 cm, respectively.

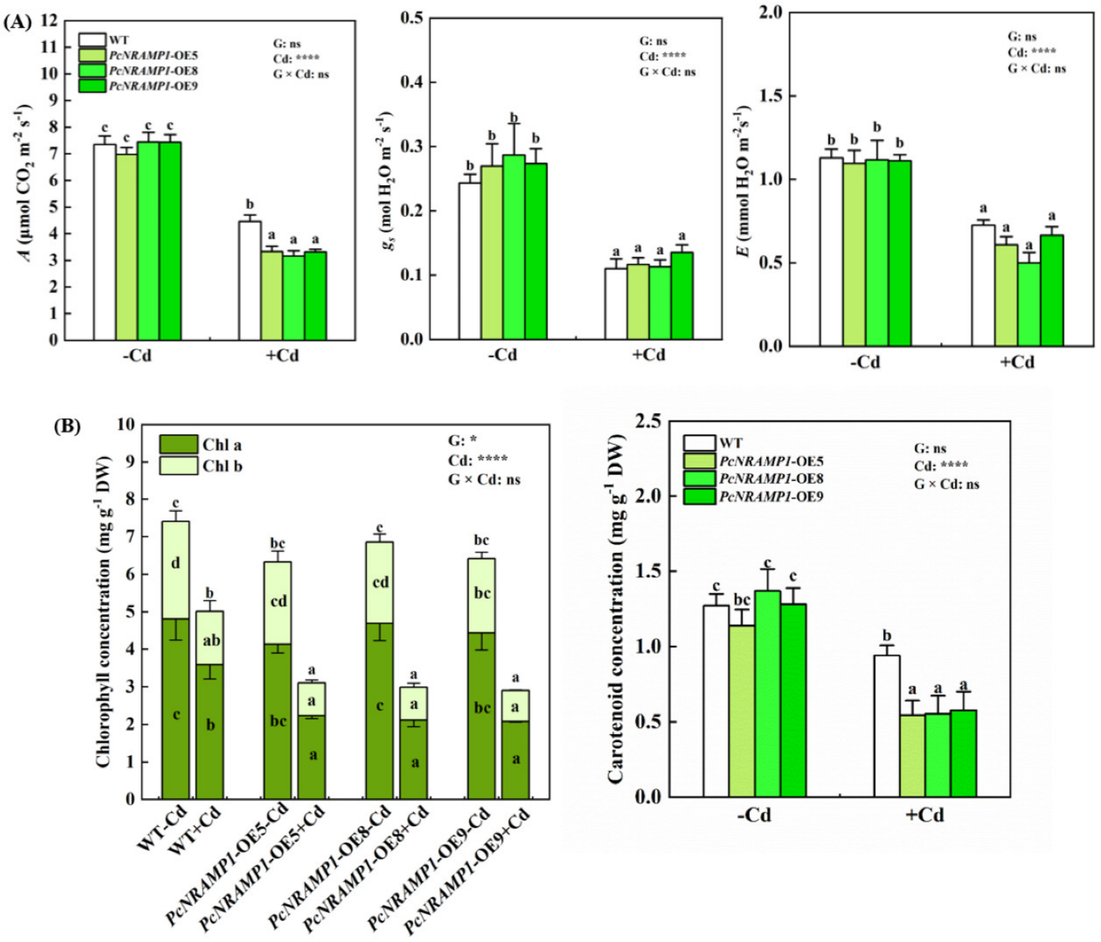

Figure S5. Photosynthesis and chlorophyll concentrations of WT and transgenic lines. (A-B) The photosynthetic characteristics and chlorophyll concentrations of WT and *PcNRAMP1*-OE5, *PcNRAMP1*-OE8, *PcNRAMP1*-OE9 of *P.* × *canescens* treated with either 0 (−Cd) or 100 (+Cd) μM $CdCl_2$ for 2 weeks. Data indicate means ± SE (n = 4). Different letters on the bars indicate significant differences between the treatments. *P*-values of the two-way ANOVAs of genotype (G), Cd and their interactions (G × Cd) are also indicated. *: *P*<0.05; **: *P*<0.01; ***: *P*<0.001; ****: *P*<0.0001; ns: not significant.
